# Field-based hyperspectral characterization of wetland plant diversity and vitality in Burullus Lagoon (Nile Delta, Egypt)

**DOI:** 10.1371/journal.pone.0341891

**Published:** 2026-03-04

**Authors:** Ghada A. Khdery, Mohamed S. Shokr, Nazih Y. Rebouh, Yasser A. El-Amier

**Affiliations:** 1 National Authority for Remote Sensing and Space Sciences (NARSS), El-Nozha El-Gedida, Cairo, Egypt; 2 Soil and Water Department, Faculty of Agriculture, Tanta University, Tanta, Egypt; 3 Department of Environmental Management, Institute of Environmental Engineering, RUDN University, Moscow, Russia; 4 Botany Department, Faculty of Science, Mansoura University, Mansoura, Egypt; Maria Curie-Sklodowska University: Uniwersytet Marii Curie-Sklodowskiej, POLAND

## Abstract

Burullus Lagoon, situated in the Nile Delta of Egypt, is a Ramsar-listed wetland of high ecological importance, particularly in relation to its floristic diversity. This study presents a field-based hyperspectral characterization of wetland vegetation with the objective of establishing a reference spectral library to support biodiversity assessment and environmental monitoring. Hyperspectral reflectance measurements were obtained for 41 plant species selected from a total of 63 floristically identified taxa, based on ecological dominance, spatial recurrence across sampling sites (≥3 stands), and suitability for reliable field spectral acquisition. Spectroscopic data were collected from 44 stands representing lagoon shores, islets, and open-water habitats using an ASD FieldSpec spectroradiometer covering the 350–2500 nm spectral range. A set of vegetation indices was applied to evaluate key biophysical and biochemical properties associated with plant vitality, water status, and biomass. The results indicate that the red and near-infrared regions provide the highest discriminatory capability among species, whereas the shortwave infrared region exhibits more limited discriminatory capability. Dominant taxa, including *Phragmites australis* and *Atriplex halimus*, displayed elevated near-infrared reflectance, consistent with differences in canopy structure and biochemical composition. Most species showed vegetation index responses broadly indicative of healthy physiological conditions, although interspecific variability suggests contrasting stress responses among taxa. Overall, the study demonstrates the applicability of field-based hyperspectral data for species-level discrimination in wetland environments and delivers a curated spectral library to support biodiversity conservation and long-term ecosystem management at Burullus Lagoon.

## 1. Introduction

Wetlands are ecologically significant environments due to their unique hydrological characteristics and their role as ecotones linking terrestrial and aquatic ecosystems [1]. Wetlands occur in a wide range of geomorphological settings, including river deltas, coastal and inland lagoons (lakes), intertidal zones, river floodplains, inland depressions, and flat terrains [2]. Egyptian coastal lakes are among the most productive wetland ecosystems worldwide. Ecologists around the world have observed and documented changes affecting these critical environments, which led to the establishment of the Ramsar Convention on Wetlands in 1971. One of the Ramsar locations, Burullus Lagoon Egypt’s deltaic Mediterranean coast, was designated as a natural protectorate in 1998 [3,4]. Burullus Lagoon, located in the Nile Delta of Egypt, is among the country’s most ecologically significant wetland systems. It serves as a vital habitat for numerous bird species and aquatic life and supports local fishing communities [5]. Plants are the primary biological components of lake ecotones. Lake ecotones often host a diverse range of plant species, including aquatic, emergent, and terrestrial vegetation [6]. Examining these plants is crucial for understanding their current status and trends, whether they are improving or deteriorating. It is essential to study these relationships to effectively manage and enhance the productivity of this area [7,8]. Over the years, human activities, pollution, and climate change have posed increasing threats to the lake’s biodiversity. To effectively study, monitor, and manage these challenges, Geographic Information System (GIS) technology has emerged as a critical tool [9]. Previous studies have shown that GIS and Remote Sensing data play significant role in assessing the ecological status of Burullus Lagoon [10–13].

Remote sensing is an efficient method for assessing plant parameters due to its global reach, cost-effectiveness, and non-destructive nature [14]. It acquires data by analyzing the Earth’s reflected and emitted waves, providing valuable information on soil, vegetation, water, and other environmental parameters with minimal effort. Remote sensing enables efficient mapping of vegetation and environmental resources, optimizing the economic potential of soils and vegetation [15]. However, most conventional remote sensing sensors acquire broadband spectral data, which may obscure narrow spectral features. Meaning that valuable information available in narrower spectral bands may be lost [16]. Previous studies have reported the use of remote sensing to assess environmental conditions in Lake Burullus Lagoon [17]. Dewidar [10] analyzed land use and land cover changes and assessed potential future changes following the construction of the international coastal road, which crosses the study area, using a Landsat image from 1984 and 1997. Dewidar and Khedr [11] determined the relationships between water quality parameters and radiance data from Landsat Thematic Mapper (TM) in Burullus Lagoon.

By analyzing multispectral or hyperspectral data, researchers can identify different types of vegetation, track their health, and assess the impact of human activities or climate change on plant cover. A hyperspectral signature refers to the unique spectral profile of an object or material, captured across a wide range of wavelengths, typically beyond the visible spectrum. Hyperspectral imaging collects detailed information by dividing the electromagnetic spectrum into hundreds of narrow bands. The hyperspectral signature of a material reveals how it absorbs, reflects, and emits light at different wavelengths, which can be used to identify and distinguish between different substances, even those that appear similar in the visible spectrum. Previous research has demonstrated the importance of hyperspectral data in providing critical information on the biochemical and biophysical characteristics of vegetation [18–23]. Advances in hyperspectral remote sensing have enhanced the accuracy of information regarding vegetation’s structural, biochemical, and physiological properties [24].

The development of new hyperspectral indices has facilitated significant progress in this field, particularly in measuring biophysical and biochemical parameters [25–27]. Spectral indices, which are mathematical transformations of spectral reflectance, are used to enhance the vegetation signal [28,29]. These hyperspectral indices offer the potential for distinguishing plant species or communities with varying canopy structures and/or biochemical compositions [30]. The vegetation in and around Burullus Lagoon plays a crucial role in maintaining the ecological balance.

Spectral analysis offers a nondestructive way to quantify plant biochemical and structural traits and to discriminate species and plant functional types [31,32]. In floristic and biodiversity studies, imaging spectroscopy links community composition with canopy chemistry and structure, supporting monitoring and conservation applications [32,33]. Vegetation indices derived from reflectance—such as NDVI, SR, and red-edge metrics—are widely used as proxies for canopy vigor, photosynthetic status, and pigment composition [34]. Recent advances also show that reflectance spectroscopy can robustly predict leaf traits across functional groups, reinforcing the physiological basis for index selection [35]. The central aim of this study is to establish a field-based hyperspectral library for Burullus Lagoon species. This library provides a baseline reference for biodiversity assessment, vegetation health evaluation, and long-term environmental monitoring. Specifically, the study investigates the spectroscopic characteristics and spectral reflectance patterns of 41 species, selected out of 63 floristically identified, and examines vegetation indices to evaluate plant vitality and canopy vigor. Accordingly, the main research question addressed in this work is whether hyperspectral signatures can reliably discriminate Burullus Lagoon plant species and their physiological conditions, thereby supporting accurate monitoring and conservation efforts.

## 2. Materials and methods

### 2.1. Study area

**Fig 1:**
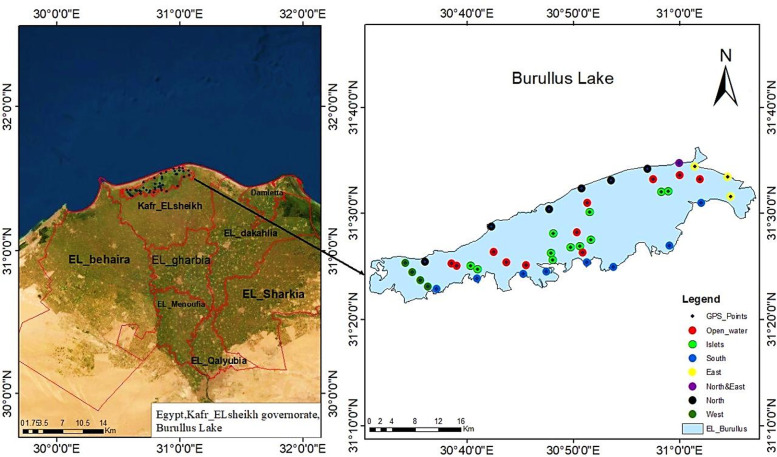
Map of the study area in middle Nile Delta showing Burullus Lagoon and sampling sites.

Burullus Lagoon, with an area of approximately 460 km^2^, is located in the Kafr El-Sheikh Governorate (30° 22’ – 31° 35’N; 30° 33’ – 31° 08’E). It lies along the eastern bank of the Nile’s Rosetta branch and is situated in the heart of the Nile Delta’s Mediterranean coast (Fig 1). The satellite image was downloaded from the USGS EarthExplorer platform (https://earthexplorer.usgs.gov) for Sentinel-2 imagery acquired in March 2022. Materials from plants, animals, or other natural environments are not collected for the study. Additionally, field site access and research operations did not require special licensing because the study was conducted to take spectroscopic measurements of plants and did not include interactions with restricted species or ecosystems. After Manzala Lake, it is Egypt’s second largest natural lake. The northern part of Kafr El-Sheikh Governorate includes El-Burullus (Baltim) district to the east, El-Hamool, El-Riad, and Sidi Salim districts to the south, and Motobas district to the west [36]. The Egyptian Environmental Affairs Agency has established and manages a network of protected sites throughout Egypt. Burullus Lagoon is one of them. It is a Ramsar site, and it has been designated as an Important Bird Area (IBA) by Birdlife International [7]. A total of forty-four geo-referenced sites were selected to represent the different habitat types (Table S1 in S2 File). The distribution of these sites is from 1 to 22 for the Lake Shores habitat, 23–33 for the Islets habitat, and 34–44 for open water (Fig 1). Fieldwork and spectral measurements were conducted between 10 and 22 March 2022, during late-winter/early-spring conditions. All sampling days experienced clear-sky weather with no rainfall events. Air temperature during measurements ranged between 22 and 28°C and relative humidity between 55 and 65%. All measurements were taken between 10:00 a.m. and 2:00 p.m. local time to minimize variation due to solar angle.

### 2.2. Floristic composition

Throughout Burullus Lagoon, 44 stands were randomly selected across three habitats: the lake shores, the lake islets, and the open water. These habitats were chosen because they represent the main ecological gradients of Burullus Lagoon —contrasting shoreline reedbeds, insular vegetation, and pelagic zones—thereby capturing the lake’s hydrological and salinity variability, in line with earlier floristic and habitat classifications [37,38]. In each stand, all plant species were recorded in five plots of 25 m^2^ each, and one representative stand, defined as a relatively homogeneous vegetation patch within each habitat [39] was used to determine species abundance (frequency, IV = 100) according to Westhoff and Van der Maarel [39] and Ellenberg [40]. The taxonomic nomenclature and identification were determined, and chorotypes were defined as the geographical distribution patterns of the recorded taxa, following the classification of Boulos (1999–2005) [41,42] and Tackholm [43]. However, life forms were identified according to Raunkiaer [44]’s system, which categorizes plants based on the position of perennating buds relative to the soil surface. In each stand, five plots of 25 m^2^ were surveyed, and species frequency and abundance were calculated as the average across all plots within the stand to ensure replication*.* This stratified random design captured the ecological heterogeneity of the lagoon, minimized sampling bias, and enhanced the statistical robustness of the floristic dataset [45]. Species abundance was calculated by averaging values from five plots within each stand to obtain representative values for frequency and abundance, ensuring that all plots contributed equally to the final calculation [39].

### 2.3. Spectroscopic measurements

Out of the 63 plant species identified during the floristic survey, a subset of 41 species was selected for hyperspectral reflectance measurements. Species selection was based on ecological relevance, spatial abundance (occurrence in ≥3 stands), and structural distinctiveness, including leaf size, canopy density, and accessibility for reliable spectroradiometric acquisition. Submerged species and morphologically indistinct taxa were excluded due to difficulties in obtaining consistent and accurate field spectra. This stratified selection ensured representative coverage of dominant and spectrally distinguishable vegetation types in Burullus Lagoon, consistent with recommendations for species-level spectral discrimination by Cochrane [46] and Clark et al., [47] on species-level spectral discrimination.

Spectral reflectance measurements were acquired using an ASD FieldSpec spectroradiometer covering the full optical spectral range from 350 to 2500 nm. The instrument provides a nominal spectral resolution of 1 nm, with sampling intervals of 1.4 nm in the 350–1050 nm range and 2 nm in the 1000–2500 nm range. All measurements were conducted under clear-sky conditions between 10:00 and 14:00 local time to minimize variations in solar angle and illumination conditions. The sensor was positioned approximately 50 cm above the canopy using a pistol grip, and measurements were restricted to mature, fully expanded, sunlit leaves to ensure spectral consistency.

For each species, three individuals were sampled to provide biological replication. For each individual, ten successive spectral scans were recorded and averaged to reduce sensor noise. Calibration was performed every 15 minutes using a Spectralon white reference panel. Raw spectra were pre-processed by removing noisy spectral regions at the edges of the spectrum (<400 nm and >2400 nm) and major atmospheric water absorption bands (1350–1450 nm and 1800–1950 nm). A two-step averaging procedure was applied, first at the scan level and subsequently at the individual level, resulting in a single representative spectrum for each species. This approach minimized within-species variability and enhanced spectral reliability for subsequent analyses.

Statistical analyses were conducted to evaluate interspecific spectral differences and classification performance. One-way analysis of variance (ANOVA) followed by Tukey’s Honestly Significant Difference (HSD) test was applied to identify spectral regions exhibiting significant interspecific variation, thereby directly testing the hypothesis of spectral separability among species. Linear Discriminant Analysis (LDA) was employed to assess species-level classification accuracy and to validate the potential of hyperspectral data for discriminating wetland vegetation in Burullus Lagoon. All statistical analyses were performed using JMP Pro software (version 14.0, SAS Institute, Cary, NC, USA).

Vegetation and sediment indices were calculated from the processed spectra using established formulations (Tables 1 and 2). Vegetation indices were selected based on their widespread use in hyperspectral studies and their ability to capture key biophysical and biochemical properties, including canopy water content, senescence, pigment composition, and red-edge dynamics. Sediment indices were included to evaluate the influence of soil salinity and water status on plant spectral responses, thereby integrating vegetation condition with habitat characteristics. The combined use of vegetation and sediment indices has been widely recommended for improving ecological interpretation and species-level discrimination in hyperspectral analyses [65].

**Table 1:** Summary of vegetation indices, equations, and sources.

Category	Index	Equation	Explanation	Source
**Canopy water content**	Moisture Stress Index	MSI = R1599/ R819	Water content	[48]
**Dry or senescent carbon**	Plant Senescence Reflectance Index	PSRI = (R680 − R500)/ R750	Chlorophyll carotenoids ratio	[49]
**Broadband greenness**	Normalized Difference Vegetation Index	NDVI = (R800 - R680)/ (R800 + R680)	Biomass content	[50–52]
	Simple Ratio	SR = R800/ R680	General plant condition	[52]
	Enhanced Vegetation Index	EVI = 2.5 × (R800 − R680)/ (R800 + 6 × R680 − 7.5 × R450 + 1)	NDVI with a correction of soil reflectance	[53]
**Leaf pigments**	Anthocyanin reflectance Index	ARI1 = 1/R550 − 1/R700	Anthocyanin amount	[54]
	Anthocyanin reflectance Index	ARI2 = R800 × (1/R550 − 1/R700)	Anthocyanin amount	[54]
	Carotenoids Reflection Index	CRI1 = (1/R510) – (1/R550)	Carotenoids/ chlorophyll ratio	[55]
	Carotenoids Reflection Index	CRI2 = (1/R510) – (1/R700)	Carotenoids/ chlorophyll ratio	[55]
	Chlorophyll a & b	Chl. a & b = (R750 - R705)/ (R750 + R705)	Leaf Chl. a & b concentrations	[56]
**Red edge vegetation indices**	Red Edge Normalized Difference Vegetation Index	RENDVI = (R750 - R705)/ (R750 + R705)	NDVI based on red edge spectral range	[57,58]
	Modified Red Edge Normalized Difference Vegetation Index	MRENDVI = (R750 − R705)/ (R750 + R705) – 2 × R445	Modification of RENDVI taking into account leaf specular reflection	[58]
	Modified Red Edge Simple Ratio (mSR705)	MRESR = (R750 − R445)/ (R705 − R445)	Red edge modification of SR.	[58,59]
	Red Edge Position Index	REPI1 = (R670 + R780)/ 2 REPI2 = 700 + 40 × ((R670 − R700)/ (R740 − R700))	Chlorophyll shifts of red edge Red-edge position (two formulations)	[60,61]

**Table 2:** Summary of sediment indices, equations and references.

Spectral Indices	Equation	Reference
Soil Adjusted Vegetation Index (SAVI)	(1 + L) (R864-R660)/ (R864 + R660 + L)	[28]
Normalized Difference Water Index (NDWI)	(R864 - R1245)/ (R864 + R1245)	[62]
Desertification Soil Index (DSI)	(R1648 – R498)/ (R1648 - R2203 + 0.2)	[63]
Salinity Index (SI)	√ R436.99 × R630.32	[64]

**Fig 2:**
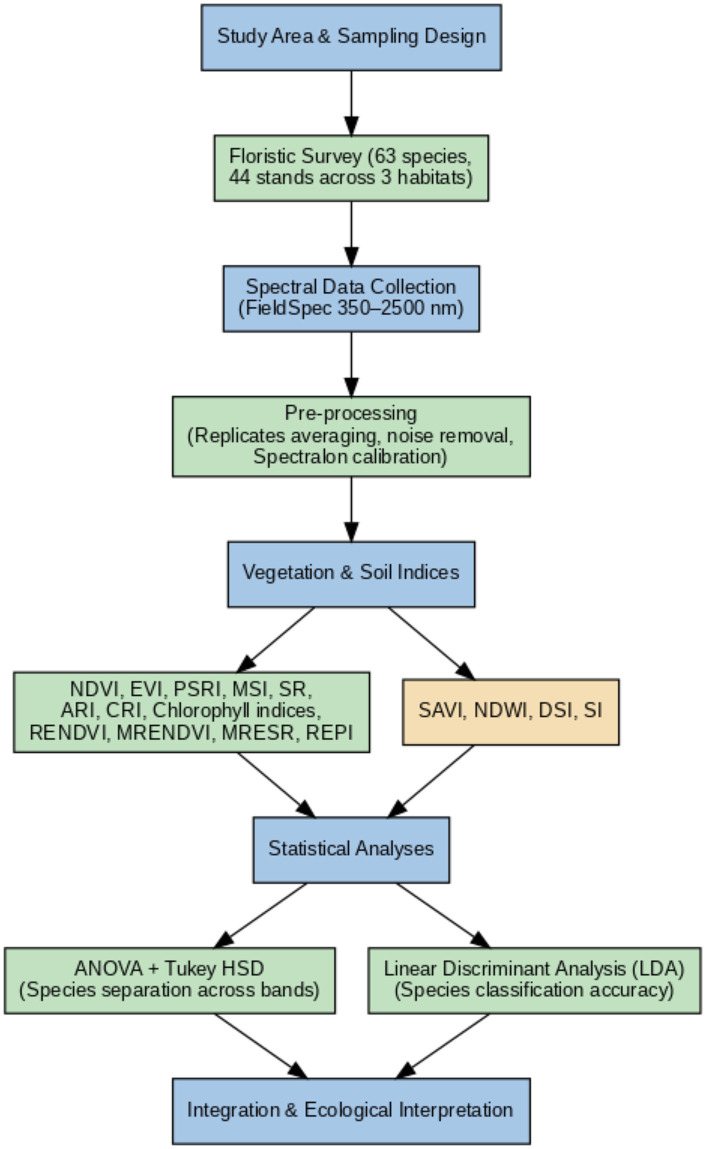
Workflow of the methodological framework applied in this study.

This workflow (Fig 2) provides a stepwise summary of the methods applied, linking field sampling, spectral data acquisition, vegetation and soil indices calculation, and statistical analyses for species discrimination and ecological interpretation.

## 3. Results

### 3.1. Floristic composition

**Fig 3:**
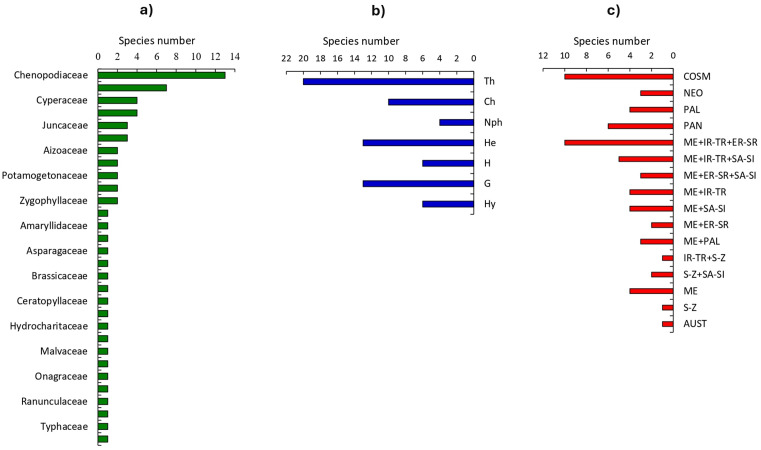
Summary of taxonomic, life-form, and chorotype patterns of the recorded species in the study area: a) species richness per family, b) distribution of plant life-forms, and c) chorotype groups.

The floristic diversity in the study area includes 63 species (19 annuals, 2 biennials, and 42 perennials) spread across 44 sites in various habitats of Burullus Lake. These species belong to 50 genera and 30 families (Table 3). The lakeshore habitat is the most floristically diverse, with 51 species, consisting of 31 perennials, 2 biennials, and 18 annual species. The flora of the lake islets habitat comprises 30 species, including 23 perennials, 1 biennial, and 6 annuals. In the open water habitat, 14 species were recorded (12 perennials and 2 annuals). Sampling was conducted during the rainy season, which favors the blooming of numerous annual species. The primary plant families include Chenopodiaceae (13 species), Asteraceae (7 species), and both Poaceae and Cyperaceae (4 species each), collectively accounting for 28 species, or 44.4% of the total species recorded. Juncaceae and Polygonaceae are represented by 3 species each, while the other 24 families were monospecific, collectively contributing 46.03% of the total species (Fig 3).

**Table 3:** The floristic composition of the different habitats in Burullus Lake.

No.	Species	Family	Life span	Life form	Chorotype	Habitat types			P%
						Lake shores	Lake islets	Open water	
**A) Hydrophytes**									
**1. Floating Hydrophytes**									
1	*Eichhornia crassipes* (C. Mart.) Solms	Pontederiaceae	Per	Hy	NEO	+	–	+	54.55
2	*Lemna gibba* L.	Lemnaceae	Per	Hy	COSM	+	–	+	15.91
3	*Ludwigia stolonifera* (Guill. & Perr.) P.	Onagraceae	Per	He	S-Z	+	–	+	18.18
4	*Marsilea aegyptiaca* Willd	Marsileaceae	Per	H, He	PAL	+	–	+	13.64
**2. Submerged hydrophytes**									
5	*Ceratophyllum demersum* L.	Ceratopyllaceae	Per	Hy	COSM	+	–	+	31.82
6	*Najas minor* All.	Hydrocharitaceae	Ann	Hy	ME + PAL	–	–	+	9.09
7	*Potamogeton crispus* L.	Potamogetonaceae	Per	Hy	PAN	+	–	+	15.91
8	*Potamogeton pectinatus*	Potamogetonaceae	Per	Hy	ME + IR-TR	+	–	+	40.91
**3. Emergent species**									
9	*Alternanthera sessilis* (L.) DC.	Amarantheceae	Per	He	PAN	+	–	–	6.82
10	*Bolboschoenus glaucus* (Lam.) S.G. Smit	Cyperaceae	Per	G, He	COSM	–	+	–	2.27
11	*Cyperus alopecuroides* Rottb.	Cyperaceae	Per	He	PAN	+	–	+	2.27
12`	*Cyperus Laevigatus* L.	Cyperaceae	Per	G, He	PAN	–	+	–	25
13	*Cyperus articulatus* L.	Cyperaceae	Per	G, He	PAN	+	–	–	2.27
14	*Echinochloa stagnina* (Retz.) P. Beauv.	Poaceae	Per	G, He	PAL	+	–	+	40.91
15	*Juncus acutus* L.	Juncaceae	Per	H	ME + IR-TR + ER-SR	+	+	–	18.18
16	*Juncus rigidus* Desf.	Juncaceae	Per	G, He	ME + IR-TR + SA-SI	+	+	–	25
17	*Juncus subulatus* Forssk.	Juncaceae	Per	G, He	ME + IR-TR + SA-SI	–	+	–	4.55
18	*Persicaria salicifolia* (Willd) Assenov	Polygonaceae	Per	G	PAL	+	–	–	2.27
19	*Phragmites australis* (Cav.) Trin. ex Steud.	Poaceae	Per	G, He	COSM	+	+	+	95.45
20	*Ranunculus sceleratus* L.	Ranunculaceae	Ann	Th	ME + IR-TR + ER-SR	+	–	+	6.82
21	*Saccharum spontaneum* L. Mant. Alt	Poaceae	Per	G, He	ME + PAL	+	–	+	20.45
22	*Typha domingensis* (Pers.) Poir.ex Steud.	Typhaceae	Per	He	PAN	+	–	+	56.82
**B) Terrestrial**									
23	*Alhagi graecorum* Boiss.	Fabaceae	Per	H	PAL	–	+	–	2.27
24	*Arthrocnemum macrostachyum* (Moric.) K. Koch	Chenopodiaceae	Per	Ch	ME + SA-SI	+	+	–	27.27
25	*Asparagus stipularis* Forssk	Asparagaceae	Per	G	ME + SA-SI	–	+	–	4.55
26	*Atriplex halimus* L.	Amaranthaceae	Per	Nph	ME + SA-SI	+	+	–	9.09
27	*Atriplex portulacoides* L.	Chenopodiaceae	Per	Ch	ME + IR-TR + ER-SR	+	+	–	18.18
28	*Atriplex prostrata* DC. in Lam.	Chenopodiaceae	Ann	Th	ME + IR-TR + ER-SR	+	+	–	11.36
29	*Atriplex semibaccata* R.Br.	Chenopodiaceae	Per	H	AUST	+	–	–	2.27
30	*Bassia indica* (Wight) A.J.Scott	Chenopodiaceae	Ann	Th	IR-TR + S-Z	+	+	–	29.55
31	*Beta vulgaris* L.	Chenopodiaceae	Bi	Th	ME + IR-TR + ER-SR	+	–	–	4.55
32	*Cakile maritima* Scop.	Brassicaceae	Ann	Th	ME + ER-SR	+	–	–	2.27
33	*Chenopodium album* L.	Chenopodiaceae	Ann	Th	COSM	+	–	–	2.27
34	*Chenopodium murale* L.	Chenopodiaceae	Ann	Th	COSM	+	+	–	15.91
35	*Cressa cretica* L.	Convolvulaceae	Per	H	ME + PAL	+	+	–	11.36
36	*Cynanchum acutum* L.	Asclepiadaceae	Per	H	ME + IR-TR	+	+	–	31.82
37	*Cynodon dactylon* (L.) Pers.	Poaceae	Per	G	COSM	+	–	–	11.36
38	*Halocnemum strobilceum* (Pallas) M. Bieb.	Chenopodiaceae	Per	Ch	ME + IR-TR + SA-SI	+	+	–	34.09
39	*Heliotropium curassavicum* L.	Boraginaceae	Per	Ch	NEO	–	+	–	4.55
40	*Limbarda crithmoides* (L.) Dumort.	Asteraceae	Per	Ch	ME + ER-SR + SA-SI	–	+	–	27.27
41	*Lactuca serriola* L.	Asteraceae	Ann	Th	ME + IR-TR + ER-SR	+	–	–	4.55
42	*Lycium schweinfurthii* Dammor	Solanaceae	Per	Nph	ME	–	+	–	4.55
43	*Malva parviflora* L.	Malvaceae	Ann	Th	ME + IR-TR	+	+	–	20.45
44	*Melilotus indicus* (L.) All.	Fabaceae	Ann	Th	ME + IR-TR + SA-SI	+	–	–	2.27
45	*Mesembryanthemum crystallinum* L.	Aizoaceae	Ann	Th	ME + ER-SR	+	–	–	6.82
46	*Mesembryanthemum nodiflorum* L.	Aizoaceae	Ann	Th	ME + ER-SR + SA-SI	+	–	–	11.36
47	*Pancratium maritimum* L.	Amaryllidaceae	Per	G	ME	–	+	–	2.27
48	*Pluchea dioscoridis* (L.) DC.	Asteraceae	Per	Nph	S-Z + SA-SI	+	–	–	6.82
49	*Polygonium equistiform* Sm.	Polygonaceae	Per	G	ME + IR-TR	+	–	–	4.55
50	*Rumex dentatus* L.	Polygonaceae	Ann	Th	ME + IR-TR + ER-SR	+	–	–	9.09
51	*Senecio glaucus* L.	Asteraceae	Ann	Th	ME + IR-TR + SA-SI	+	+	–	6.82
52	*Silybium marianum* (L.) Gaertn.	Asteraceae	Ann	Th	ME + IR-TR + ER-SR	+	–	–	4.55
53	*Solanum nigrum* L.	Solanaceae	Ann	Th	COSM	+	–	–	2.27
54	*Sonchus oleraceus* L.	Asteraceae	Ann	Th	COSM	+	–	–	11.36
55	*Spergularia marina* (L.) Griseb.	Caryophyllaceae	Bi	Th	ME + IR-TR + ER-SR	+	+	–	11.36
56	*Suaeda maritima* (L.) Dumort	Chenopodiaceae	Ann	Th	COSM	+	+	–	6.82
57	*Suaeda pruinosa* Lange	Chenopodiaceae	Per	Ch	ME	+	+	–	11.36
58	*Suaeda vera* Forssk. ex J.F. Gmel.	Chenopodiaceae	Per	Ch	ME + ER-SR + SA-SI	–	+	–	4.55
59	*Symphyotrichum squamatum* (Spren.) Nesom	Asteraceae	Per	Ch	NEO	+	+	–	18.18
60	*Tamarix nilotica* (Ehrenb.) Bunge	Tamaricaceae	Per	Nph	S-Z + SA-SI	+	+	–	29.55
61	*Urtica urnes* L.	Urticaceae	Ann	Th	ME + IR-TR + ER-SR	+	–	–	6.82
62	*Zygophyllum aegyptium* Hosny	Zygophyllaceae	Per	Ch	ME	+	–	–	4.55
63	*Zygophyllum album* L.	Zygophyllaceae	Per	Ch	ME + SA-SI	–	+	–	2.27
**Number of stands**					**44**	**22**	**11**	**11**	
**Number of perennials**					**42**	**31**	**23**	**12**	
**Number of biennials**					**2**	**2**	**1**	**0**	
**Number of annuals**					**19**	**18**	**6**	**2**	
**Total number of recorded species**					**63**	**51**	**30**	**14**	

***Abbreviations:* P:** Presence, **Life-span; Per:** Perennials, Bi: Biennials, **Ann**: Annuals**, Life-form; Nph**: Nanophanerophytes, **Ch**: Chamaephytes, **H:** Hemicryptophytes, **G**: Geophytes, **He**: Helophytes, **Hy**: Hydrophytes, **Th**: Therophytes, **Chorotype;** COSM: Cosmopolitan, PAN: Pantropical, NEO: Neotropical, PAL: Palaeotropical, IR-TR: Irano-Turanian, S-Z: Sudano-Zambezian, Cult. & Nat.: Cultivated and Naturalized, ME: Mediterranean, ER-SR: Euro-Siberian, SA-SI: Saharo-Sindian.

The broad ecological range of Chenopodiaceae and Asteraceae, spanning saline coastal regions, deserts, temperate grasslands, and disturbed sites, is due to several adaptive traits that enable them to thrive in diverse and often challenging environments (Fig 3). Chenopodiaceae species are typically halophytic, with a high salinity tolerance, allowing them to colonize areas unsuitable for many other plant families [66]. Asteraceae species are highly drought-resistant, and their effective wind dispersal of diaspores aids their survival [67]. Poaceae have developed traits that help them endure and even benefit from grazing and drought, such as an extensive shallow root system that efficiently absorbs soil moisture [68]. Cyperaceae species are particularly adapted to wetlands, marshes, and waterlogged areas, with many sedges thriving in both aquatic and terrestrial environments, making them common in areas with varying water levels [69].

Based on their percentage presence, the recorded species were classified into three main categories: 1) the wide-range distribution class (presence > 50%), consisting of three species: *Phragmites australis* (95.45%), *Typha domingensis* (56.82%), and *Eichhornia crassipes* (54.55%); 2) the moderate distribution class (presence between 25–50%), which includes 11 species such as *Echinochloa stagnina* and *Potamogeton pectinatus* (both at 40.91%), *Halocnemum strobilceum* (34.09%), *Ceratophyllum demersum* and *Cynanchum acutum* (both at 31.82%); 3) the narrow-range distribution class (presence < 25%), which includes 49 species, such as *Saccharum spontaneum* (20.45%), *Lemna gibba* and *Potamogeton crispus* (both at 15.91%), *Atriplex prostrata* (11.36%), and *Alternanthera sessilis* (6.82%) (Table 3).

The life-form spectrum in the study area and its various habitats is predominantly composed of cryptophytes, which include helophytes, geophytes, and hydrophytes, with additional representation from therophytes, chamaephytes, and hemicryptophytes. Nanophanerophytes are the least represented group across all habitats in the study area (Fig 3b). Cryptophytes account for 32% of the life forms in the overall study area, with values of 23%, 12%, and 16% in lake shores, lake islets, and open-water habitats, respectively. This life form contributed about 20.5%, 20.9%, 26.3%, and 28.57% in the studies of Al-Sodany [70], and, Shaltout et al., [71] respectively. Therophytes represent approximately 20% of the species in the study area and are distributed as 20%, 7%, and 1% in lake Shores, lake Islets, and open water, respectively. Therophytes dominate due to topographical changes and human or animal disturbances [72]. Their ability to survive as seeds during dry periods makes them well-adapted to the region’s arid conditions. These results are consistent with plant life forms in arid environments across the Middle East [73]. Moreover, Chamaephytes make up 10% of the species in the study area, with 6% in Lake Shores and 9% in Lake Islets. Many chamaephytes possess adaptations that allow them to tolerate saline conditions, which are common in coastal and brackish environments like Burullus Lake. Their ability to survive in areas with high salinity provides them a competitive advantage over other plant forms [74].

### 3.2. Chorological affinities

Egypt serves as a crossroads for floristic components from four phytogeographical regions: African Sudano-Zambian, Asiatic Irano-Turanian, Afro-Asian Sahro-Sindian, and Euro-Afro-Asian Mediterranean [75]. The entire country falls within the Saharo-Arabian belt of the Holarctic floristic region. The chorological analysis of the area indicates that 35 species, approximately 55.55% of the total recorded species, are Mediterranean taxa (Fig 3. 2c). These include 18 plurinational species (28.57%), 16 biregional species (25.39%), and 6 mono regional species (9.52%). Additionally, 23 species, around 36.50% of the total, are worldwide species, categorized as 10 cosmopolitan (15.87%), 4 palaeotropical (6.35%), 6 pantropical (9.52%), and 3 neotropical (4.76%) (Table 3). Other floristic categories are underrepresented, with each chorotype consisting of only a few species. In terms of habitats, Mediterranean elements are most abundant in lake shores (27 taxa), followed by lake islets (20 taxa), and then open water (4 taxa) (Table 3).

### 3.3. Spectral analysis

#### 3.3.1. Spectroscopic parameters and analysis.

**Fig 4:**
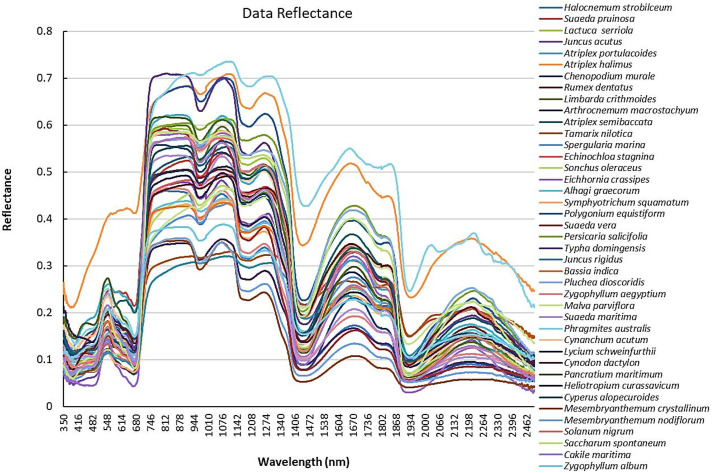
Spectral reflectance pattern for plant species.

Analysis of the spectroscopic parameters revealed that all plant species exhibit a consistent spectral shape across various spectral zones, particularly in the visible and infrared regions [18,20,22]. The spectral signature shows that the highest reflectance was observed in the NIR zone between (944–1076 nm) while the lowest reflectance was in SWIRII zone from (2400–2462 nm) (Supplementary Data S1 in S1 File). Moderate reflectance was found in visible region from (555–614 nm) and SWIRI zone from (1538–1670 nm) (Fig 4). *Phragmites australis* and *Atriplex halimus* exhibited higher spectral reflectance than the other species in all spectral zones, whereas *Phragmites australis* showed close spectral reflectance pattern to *Atriplex halimus*. *Mesembryanthemum crystallinum* showed the lowest reflectance in SWIRII while Juncus rigidus showed the lowest reflectance in NIR. In the visible region, reflectance was largely convergent among species, with limited interspecific differentiation. In visible zone, *Cakile maritima* showed a relatively different reflectance pattern [76].

#### 3.3.2. Statistical analyses including Tukey’s HSD and LDA.

**Fig 5:**
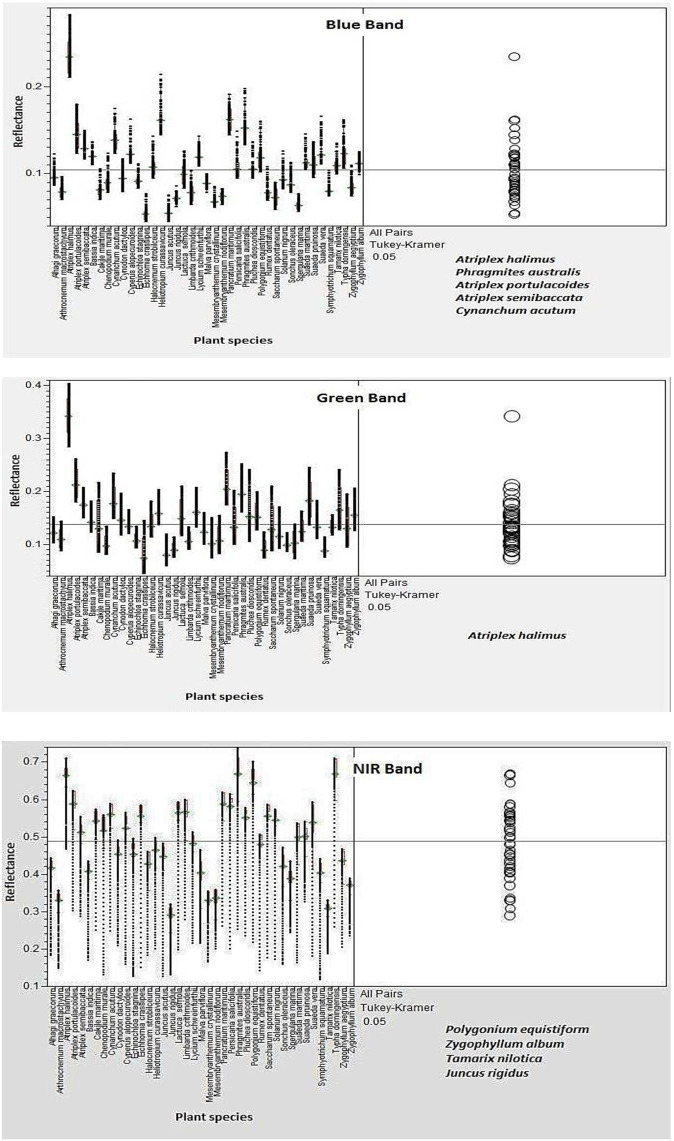
ANOVA and Tukey HSD results for differentiating plant samples across the blue, green and NIR spectral Zones.

**Fig 6:**
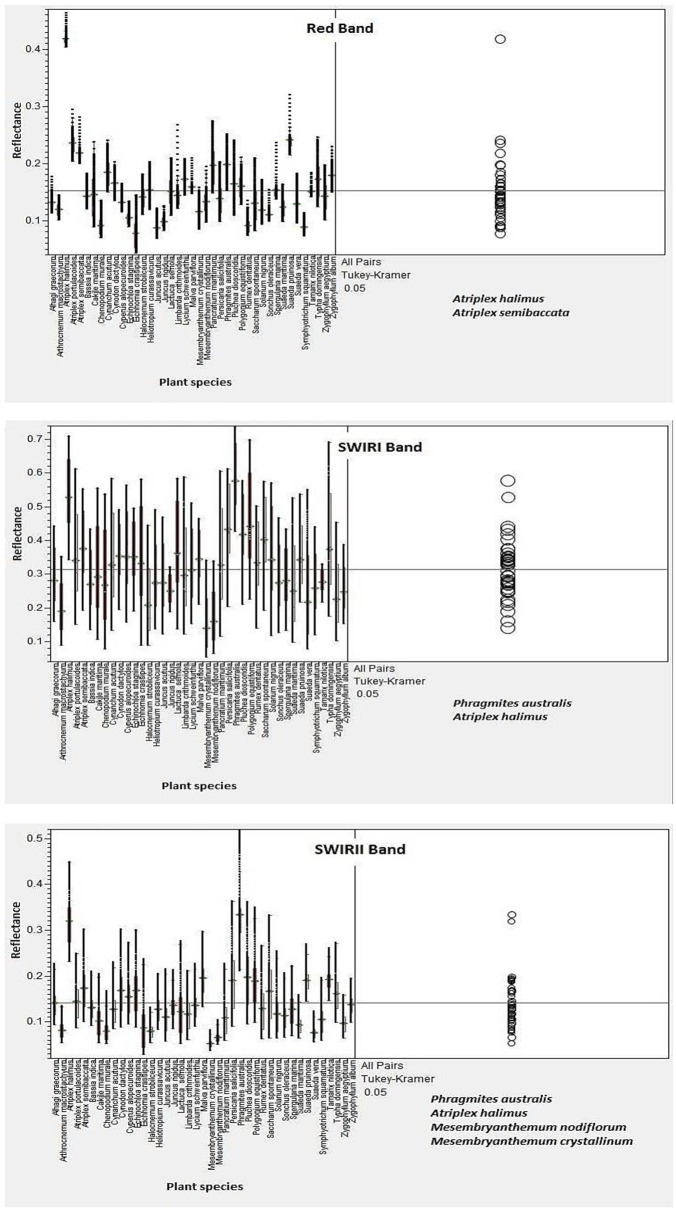
ANOVA and Tukey HSD analyses for differentiating plant samples across Red, SWIR I and SWIR II spectral Zones.

To address our aim of discriminating taxa and identifying the most informative wavebands, we used band-wise ANOVA/Tukey to test interspecific separability across spectral regions and LDA to quantify species-level classification accuracy. Prior to these analyses, the standard deviation (STDV) of reflectance was computed for each species from its three field replicates to evaluate within-species spectral variability. The complete STDV dataset for all 41 taxa is presented in Table S2 in S2 File and was used to confirm the consistency of spectral replicates and ensure that interspecific differences detected by ANOVA and LDA reflected true taxonomic separation rather than within-species variability. A one-way ANOVA followed by Tukey’s Honestly Significant Difference (HSD) post-hoc tests was conducted across the major spectral regions blue, green, and red (Fig 5) and NIR, SWIR I, and SWIR II (Fig 6) to assess interspecific spectral difference. The results showed that the Red (600–700 nm) and NIR (750–900 nm) bands provided the strongest discriminatory power among species, with highly significant F-values (p < 0.001). In contrast, reflectance differences in the green region (500–600 nm) were generally non-significant, indicating limited value for species-level separation. This non-significance in the green band is expected because reflectance near ~550 nm is dominated by broad chlorophyll absorption and multiple scattering, which reduces interspecific contrast compared with the red edge and NIR. Tukey’s grouping further indicated that dominant taxa such as *Phragmites australis*, *Typha domingensis*, and *Atriplex halimus* formed significantly distinct clusters (p < 0.05), whereas halophytic species such as *Suaeda spp.* displayed overlapping groups, suggesting partial spectral convergence under saline conditions (Table 4).

**Table 4:** One-way ANOVA and Tukey HSD results for species discrimination across spectral regions.

Spectral zone	F-value	df	p-value	η² (effect size)	Tukey grouping
Red (600–700 nm)	32.7	40, 160	<0.001	0.89	*Phragmites australis* & *Atriplex halimus* (high); *Juncus rigidus* & *Cakile maritima* (low)
Green (500–600 nm)	3.9	40, 160	0.072	0.49	Not significant
NIR (750–900 nm)	47.9	40, 160	<0.001	0.92	*Phragmites australis* & *Typha domingensis* (high); *Suaeda monoica* & *Juncus rigidus* (low)
SWIR-I (1500–1700 nm)	8.5	40, 160	0.004	0.68	Moderate separation
SWIR-II (2000–2400 nm)	25.6	40, 160	<0.001	0.86	*Mesembryanthemum crystallinum* (low); *Eichhornia crassipes* (high)

Effect sizes (η²) were calculated for each spectral region to complement the ANOVA results and quantify the magnitude of interspecific spectral differences. The Red and NIR regions exhibited very large effect sizes (η² = 0.89 and 0.92), indicating strong discriminatory power, while SWIR-I and SWIR-II showed moderate-to-high effects (η² = 0.68 and 0.86). In contrast, the Green region exhibited a weaker, non-significant effect (η² = 0.49). These values are included in the revised ANOVA Table 4. Linear Discriminant Analysis (LDA) explained 89.4% of the total variance within the first two canonical discriminant functions. The overall classification accuracy reached 86.5% at the species level. Dominant species such as *Phragmites australis*, *Typha domingensis*, and *Eichhornia crassipes* were correctly separated with >90% accuracy, while species with subtler spectral differences (*Juncus rigidus*, *Suaeda monoica*) exhibited moderate misclassification. The confusion matrix (Table 5) highlights that dominant taxa were consistently well-separated, whereas morphologically and spectrally similar species showed partial overlap. Misclassifications mainly occurred among morphologically and biochemically similar halophytes (e.g., *Juncus* spp., *Suaeda*–*Spergularia*), consistent with convergent stress-adapted pigment signatures. Validation was performed using leave-one-out cross-validation (LOOCV), which produced stable classification performance with only a slight reduction in overall accuracy. Producer’s accuracy values ranged from 74% to 93%, while user’s accuracy ranged from 71% to 95%, indicating strong separability for dominant species and moderate overlap among morphologically similar halophytes. The confusion matrix further confirmed these trends by showing high correct-classification rates for emergent macrophytes and limited confusion among stress-adapted chenopodiaceous species.

**Table 5:** Linear Discriminant Analysis (LDA) classification results for dominant wetland species based on spectral signatures.

Species	Correctly classified (%)	Misclassified with
*Phragmites australis*	94.1	–
*Typha domingensis*	92.3	*P. australis* (7.7%)
*Eichhornia crassipes*	90.5	*Cyperus alopecuroides* (9.5%)
*Atriplex halimus*	89.7	*Suaeda vera* (10.3%)
*Spergularia marina*	87.2	*Suaeda monoica* (12.8%)
*Juncus rigidus*	76.4	*Juncus acutus* (23.6%)
*Suaeda monoica*	74.8	*Spergularia marina* (25.2%)
*Cakile maritima*	71.5	*Sonchus oleraceus* (28.5%)
Other taxa (average, n = 33)	82.6	Variable

#### 3.3.3. Wavelength of plants samples.

The findings highlight the optimal wavebands suitable for identifying the studied plant species (Table 6). Overall, 12 species were found to have only a single narrow spectral zone (e.g., *Arthrocnemum macrostachyum*, *Cakile maritima*, *Cyperus alopecuroides*, and *Tamarix nilotica*), which limits their separability to specific wavelengths. In contrast, two species, *Atriplex halimus* and *Spergularia marina*, exhibited exceptionally broad spectral ranges that extended across the visible, NIR, and SWIR domains, demonstrating their high diagnostic potential.

**Table 6:** Optimal spectral wavelengths and zones for discriminating plant species in Burullus wetland.

Plant Name	Optimal Wavelength	Spectral Range
*Alhagi graecorum* Boiss.	591-632-837-878-960-1001	Red – NIR
*Arthrocnemum macrostachyum* (Moric.) K. Koch	871-912	NIR
*Atriplex halimus* L.	375-416-457-498-539-580-621-662-703-744-785-826-867-908-949-990-1031-1072-1113-1154-1195-1236-1277-1318-1359-1400-1441-1482-1523-1564-1605-1646-1687-1728-1769-1810-1851-1892-1933-1974-2015-2056-2097-2138-2179-2220-2261-2302-2343-2384-2425-2466	Blue – Green – Red – NIR – SWIRI – SWIRII
*Atriplex semibaccata* R.Br.	380-421-462-503-626-667	Blue – Green – Red
*Atriplex portulacoides* L.	–	–
*Bassia indica* (Wight) A.J.Scott	598-885-1049	Red – NIR
*Cakile maritima* Scop.	614-655-696	Red
*Chenopodium murale* L.	–	–
*Cynanchum acutum* L.	973-1014-1055-1096-1178-1219-1342-1506	NIR – SWIRI
*Cynodon dactylon* (L.) Pers.	483-852	Green – NIR
*Cyperus alopecuroides* Rottb.	1019	NIR
*Echinochloa stagnina* (Retz.) P. Beauv.	752	NIR
*Eichhornia crassipes* (C. Mart.) Solms	631-672-1041-1410-1615-1820-1861-1984-2025-2107-2148-2189-2271-2312-2353-2394-2435-2476	Red – NIR – SWIRI – SWIRII
*Halocnemum strobilceum* (Pallas) M. Bieb.	903-1067-1190-1231	NIR – SWIRI
*Heliotropium curassavicum* L.	1346-1387-1428-1469-1510-1551	SWIRI
*Juncus acutus* L.	578-619-660-701-742-783-824-865-906-947-988-1029	Red – NIR
*Juncus rigidus* Desf.	761-802-843-884-1909-2032-2114-2155-2196	NIR – SWIRII
*Lactuca serriola* L.	864-905	NIR
*Limbarda crithmoides* (L.) Dumort.	911	NIR
*Lycium schweinfurthii* Dammor	851	NIR
*Malva parviflora* L.	437-847	Blue – NIR
*Mesembryanthemum crystallinum* L.	569-651-692-774-815-856-897-938-979-1061-1102-2373	Red – NIR -SWIRI – SWIRII
*Mesembryanthemum nodiflorum* L.	365-406-488-2456-2497	Blue – Green-SWIRII
*Pancratium maritimum* L.	361-402-443-484-525-566-607-648-689-730-771-812-853-894	Blue – Green-Red – NIR
*Persicaria salicifolia* (Willd) Assenov	1292-1333-1374-1415-1456-1497-1538	SWIRI
*Phragmites australis* (Cav.) Trin.ex	1095-1136-1177-1218-1259-1341-1382	NIR – SWIRI
*Pluchea dioscoridis* (L.) DC.	1091-1132	NIR – SWIRI
*Polygonium equistiform* Sm.	1085-1167	NIR – SWIRI
*Rumex dentatus* L.	1156-1279-1361-1402-1443-1566	SWIRI
*Saccharum spontaneum* L. Mant. Alt	–	–
*Solanum nigrum* L.	–	–
*Sonchus oleraceus* L.	1122-1163-1204-1245-1286-1327-1450-1491	SWIRI
*Spergularia marina* (L.) Griseb.	423-464-587-628-669-710-751-792-833-874-997-1038-1120-1161-1202-2022-2063-2104-2145-2186-2227-2268-2350-2391-2432-2473	Blue – Green –Red – NIR –SWIRI – SWIRII
*Suaeda maritima* (L.) Dumort	1053-1217-1258	NIR – SWIRI
*Suaeda pruinosa* Lange	576-617-822	Red – NIR
*Suaeda vera* Forssk. ex J.F. Gmel.	1086-1209-1250	NIR – SWIRI
*Symphyotrichum squamatum* (Spren.) Nesom	920-1002-1084-1207-2150	NIR – SWIRI -SWIRII
*Tamarix nilotica* (Ehrenb.) Bunge	955-996	NIR
*Typha domingensis* (Pers.) Poir.ex Steud.	1088-1129-1211	NIR – SWIRI
*Zygophyllum aegyptium* Hosny	600-641-764-928-969-1051-1092-1133-1174	Red – NIR -SWIRI
*Zygophyllum album* L.	–	–

Among the spectral regions, the red (600–700 nm) and NIR (750–900 nm) zones were the most informative, covering the largest number of distinctive taxa (23 and 19 species, respectively). By contrast, the SWIR-I and SWIR-II ranges, though less represented overall, were critical for discriminating halophytic species such as *Mesembryanthemum crystallinum* and *Spergularia marina*, which showed unique reflectance beyond 2000 nm. The Green zone (500–600 nm) contributed only marginally, with fewer taxa showing separability.

Empty cells in Table 6 indicate species for which no unique diagnostic wavelengths could be identified, reflecting either overlapping spectral patterns with other taxa (e.g., *Chenopodium murale*, *Saccharum spontaneum*, *Solanum nigrum*, *Zygophyllum album*) or insufficient distinctiveness in their reflectance curves.

These results emphasize that while red and NIR regions remain the most reliable for species-level discrimination in Burullus wetland vegetation, the SWIR bands provide valuable complementary information for halophytes and broad-spectrum species.

#### 3.3.4. Hyperspectral vegetation indices.

A series of vegetation indices derived from spectrometric analysis were employed to assess the vitality of the plants, as detailed in Table 7. The interpretation of vegetation indices provided critical insights into plant physiological status. NDVI values below 0.5 are widely accepted as indicators of stressed or sparse vegetation, while values above 0.6 reflect dense and healthy canopies [77].In this study, most species, such as *Atriplex halimus*, *Tamarix nilotica*, and *Suaeda pruinosa* recorded low NDVI values (<0.5), reflecting moderate stress and reduced biomass. By contrast, *Eichhornia crassipes* (0.85) and *Chenopodium murale* (0.76) indicated vigorous growth, consistent with healthy vegetation thresholds [78].

**Table 7:** Vegetation index values (MSI, NDVI, PSRI, SR, and EVI).

No.	Plant Species	MSI	NDVI	PSRI	SR	EVI
1	*Halocnemum strobilceum* (Pallas) M. Bieb.	0.3330	0.6097	−0.0163	4.13	1.55
2	*Suaeda pruinosa* Lange.	0.5722	0.3942	0.1028	2.31	2.67
3	*Lactuca serriola* L.	0.5285	0.6819	−0.0299	5.28	2.19
4	*Juncus acutus* L.	0.4764	0.7385	0.0081	6.65	2.12
5	*Atriplex portulacoides* L.	0.4554	0.4993	0.0122	2.99	2.29
6	*Atriplex halimus* L.	0.7329	0.2325	0.1179	1.61	3.19
7	*Chenopodium murale* L.	0.3585	0.7657	−0.0173	7.54	2.13
8	*Rumex dentatus* L.	0.6092	0.7239	0.0057	6.25	2.09
9	*Limbarda crithmoides* (L.) Dumort.	0.3803	0.6545	0.0500	4.79	2.84
10	*Arthrocnemum macrostachyum* (Moric.) K. Koch	0.4388	0.5427	0.0025	3.37	1.37
11	*Atriplex semibaccata* R.Br.	0.6552	0.4345	0.0671	2.54	2.27
12	*Tamarix nilotica* (Ehrenb.) Bunge.	0.8408	0.3657	0.0560	2.15	1.13
13	*Spergularia marina* (L.) Griseb.	0.6492	0.4461	0.1391	2.61	2.33
14	*Echinochloa stagnina* (Retz.) P. Beauv.	0.6720	0.6723	−0.0106	5.11	1.72
15	*Sonchus oleraceus* L.	0.5391	0.6084	0.0267	4.11	1.75
16	*Eichhornia crassipes* (C. Mart.) Solms.	0.4858	0.8496	−0.0044	12.29	2.64
17	*Alhagi graecorum* Boiss.	0.5443	0.5746	0.0093	3.71	1.67
18	*Symphyotrichum squamatum* (Spren.) Nesom.	0.5118	0.6957	−0.0069	5.57	1.63
19	*Polygonium equistiform* Sm.	0.5518	0.6709	−0.0082	5.07	2.59
20	*Suaeda vera* Forssk. ex J.F. Gmel.	0.2364	0.7135	−0.0269	5.98	2.07
21	*Persicaria salicifolia* (Willd) Assenov.	0.6615	0.7121	−0.0131	5.95	2.39
22	*Typha domingensis* (Pers.) Poir.ex Steud.	0.4172	0.6924	−0.0154	5.51	2.77
23	*Juncus rigidus* Desf.	0.7837	0.5446	0.0069	3.39	1.05
24	*Bassia indica (Wight) A.J.Scott.*	0.5485	0.5527	−0.0161	3.47	1.31
25	*Pluchea dioscoridis (L.) DC.*	0.6906	0.6673	−0.0172	5.01	2.27
26	*Zygophyllum aegyptium Hosny.*	0.3730	0.6326	−0.0099	4.44	1.87
27	*Malva parviflora* L.	0.7987	0.4382	0.0978	2.56	1.92
28	*Suaeda maritima* (L.) Dumort.	0.3416	0.6797	−0.0167	5.24	1.93
29	*Phragmites australis* (Cav.)Trin.ex.	0.7722	0.6235	−0.0363	4.32	2.25
30	*Cynanchum acutum* L.	0.4538	0.5838	−0.0076	3.81	2.06
31	*Lycium schweinfurthii* Dammor.	0.5308	0.5441	−0.0016	3.38	1.83
32	*Cynodon dactylon* (L.) *Pers.*	0.6929	0.5443	0.0082	3.39	1.81
33	*Pancratium maritimum* L.	0.4239	0.6084	−0.0477	4.11	1.65
34	*Heliotropium curassavicum* L.	0.4600	0.5968	−0.0399	3.96	1.23
35	*Cyperus alopecuroides* Rottb.	0.5551	0.6486	−0.0119	4.69	1.92
36	*Mesembryanthemum crystallinum* L.	0.2643	0.6011	0.0033	4.02	1.47
37	*Mesembryanthemum nodiflorum* L.	0.3322	0.5589	0.0237	3.53	1.47
38	*Solanum nigrum* L.	0.5155	0.7117	−0.0007	5.94	2.28
39	*Saccharum spontaneum* L. Mant. Alt.	0.6389	0.7510	−0.0314	7.03	2.34
40	*Cakile maritima* Scop.	0.4004	0.7185	−0.0133	6.11	2.41
41	*Zygophyllum album L.*	0.5581	0.4291	0.0290	2.51	1.45

PSRI values between –0.1 and 0.2 are generally associated with healthy vegetation, with higher values reflecting increasing pigment degradation or senescence [49,58]. The species examined here exhibited PSRI values within this healthy range (–0.04 to 0.14). However, species such as *Spergularia marina* (0.14) and *Atriplex halimus* (0.12) displayed relatively higher values, suggesting the onset of pigment degradation, consistent with early senescence.

The Moisture Stress Index (MSI) is positively correlated with canopy water stress, with values >0.7 indicating limited leaf water content and higher stress levels [65]. In the present dataset, species such as *Tamarix nilotica* (0.84), *Malva parviflora* (0.79), and *Phragmites australis* (0.77) showed evidence of water stress. In contrast, low MSI values in *Suaeda vera* (0.23) confirmed a healthy water status.

The Simple Ratio (SR) is another widely used broadband index, where values above 3 are indicative of good canopy condition and higher chlorophyll concentration. In this study, most species recorded SR > 3, confirming generally good physiological condition. Exceptions included *Atriplex halimus* (1.61) and *Tamarix nilotica* (2.15), which exhibited lower SR values consistent with their elevated MSI and reduced NDVI, highlighting multi-index agreement in identifying stressed vegetation.

To strengthen ecological interpretation, species-level examples were incorporated based on established spectral thresholds. For instance, high NDVI and low PSRI values in *Phragmites australis* indicate dense, vigorous reed stands; moderate NDVI in *Typha domingensis* reflects partial senescence; and low NDVI combined with higher stress indices (MSI, PSRI) in *Juncus acutus* is consistent with salinity-induced stress and sparse canopy structure [79].

Together, these indices demonstrate that vegetation in Burullus Lagoon is highly heterogeneous, with halophytes such as *Atriplex halimus* and *Tamarix nilotica* showing clear signs of stress, while aquatic macrophytes such as *Eichhornia crassipes* maintained vigorous growth. The integration of NDVI, PSRI, MSI, and SR allowed for cross-validation of stress signals, strengthening the interpretation of hyperspectral measurements. Ecologically, these multi-index patterns indicate spatial mosaics of productivity and stress across habitats, supporting targeted monitoring of invasive hydrophytes and salt-tolerant stands along the lagoon’s salinity and water-level gradients.

Because field measurements were taken under clear-sky, midday conditions, most taxa were sampled during early to mid-growing stages; thus, higher NDVI and chlorophyll-related indices in hydrophytes (e.g., *Eichhornia crassipes*) and lower values in halophytes (e.g., *Atriplex halimus*, *Tamarix nilotica*) likely reflect phenology interacting with water availability and irradiance. This seasonal context helps explain why red-edge indices (RENDVI, REPI) were especially diagnostic, capturing chlorophyll build-up in actively growing canopies while flagging pigment loss in stress-prone taxa.

Analysis of pigment-related indices provided additional biochemical insights into species’ physiological responses. Pigment indices (ARI, CRI) add biochemical context to structural indices: high ARI suggests stress-linked anthocyanins, while high CRI indicates carotenoid-based photoprotection (Table 8). The Anthocyanin Reflectance Index (ARI) revealed notable variation across taxa. Most species recorded ARI1 values close to zero, consistent with limited anthocyanin accumulation. However, *Limbarda crithmoides* (ARI1 = 3.80), *Spergularia marina* (3.06), and *Mesembryanthemum nodiflorum* (1.31) exhibited markedly high ARI values, indicating anthocyanin accumulation as a stress-response mechanism. Elevated ARI is widely recognized as a marker of photoprotection and abiotic stress adaptation [80,81].

**Table 8:** Calculated Leaf Pigment Index: Carotenoid Reflectance Index (CRI 1 & 2), Anthocyanin Reflectance Index (ARI & 2) and chlorophyll a and b concentration.

No.	Plant Species	Leaf pigment Index				
Anthocyanin Reflectance Index		Carotenoids Reflectance Index		Leaf Chlorophyll a and b Concentration
ARI1	ARI2	CRI1	CRI2	Chl. a & b
1	*Halocnemum strobilceum* (Pallas) M. Bieb.	−0.1315	−0.0603	2.27	2.15	0.3544
2	*Suaeda pruinosa* Lange.	0.9468	0.4738	1.34	2.29	0.1516
3	*Lactuca serriola* L.	−0.5037	−0.2982	2.14	1.64	0.4288
4	*Juncus acutus* L.	0.1500	0.0703	4.98	5.14	0.4852
5	*Atriplex portulacoides* L.	0.4368	0.2693	0.87	1.32	0.2819
6	*Atriplex halimus* L.	0.3216	0.2134	0.36	0.68	0.1367
7	*Chenopodium murale* L.	−0.6650	−0.3706	3.66	2.99	0.5464
8	*Rumex dentatus* L.	0.6960	0.3464	3.83	4.53	0.4679
9	*Limbarda crithmoides* (L.) Dumort.	3.8023	2.2558	2.07	5.87	0.2871
10	*Arthrocnemum macrostachyum* (Moric.) K. Koch	0.0774	0.0266	2.15	2.23	0.3316
11	*Atriplex semibaccata* R.Br.	1.2920	0.6611	0.78	2.07	0.2217
12	*Tamarix nilotica* (Ehrenb.) Bunge.	0.9762	0.2992	1.16	2.14	0.1854
13	*Spergularia marina* (L.) Griseb.	3.0673	1.1568	2.09	5.16	0.1428
14	*Echinochloa stagnina* (Retz.) P. Beauv.	−0.6473	−0.3031	2.24	1.59	0.5030
15	*Sonchus oleraceus* L.	1.7347	0.7217	2.05	3.79	0.3618
16	*Eichhornia crassipes* (C. Mart.) Solms.	−0.2602	−0.1506	9.07	8.82	0.4754
17	*Alhagi graecorum* Boiss.	0.9410	0.4025	1.68	2.63	0.3343
18	*Symphyotrichum squamatum* (Spren.) Nesom.	−0.0515	−0.0217	3.10	3.05	0.4871
19	*Polygonium equistiform* Sm.	0.2924	0.1940	1.72	2.01	0.4246
20	*Suaeda vera* Forssk. ex J.F. Gmel.	−0.3235	−0.1913	2.53	2.21	0.4540
21	*Persicaria salicifolia* (Willd) Assenov.	−0.3202	−0.1915	3.16	2.84	0.4073
22	*Typha domingensis* (Pers.) Poir.ex Steud.	0.0769	0.0545	2.22	2.29	0.3853
23	*Juncus rigidus* Desf.	0.8476	0.2392	2.64	3.48	0.2863
24	*Bassia indica* (Wight) A.J.Scott.	−0.5437	−0.2291	1.85	1.31	0.3588
25	*Pluchea dioscoridis* (L.) DC.	−0.3070	−0.1737	2.69	2.38	0.3400
26	*Zygophyllum aegyptium* Hosny.	0.0439	0.0203	2.78	2.83	0.3150
27	*Malva parviflora* L.	1.5208	0.5829	1.77	3.29	0.2082
28	*Suaeda maritima* (L.) Dumort.	−0.2450	−0.1304	2.25	2.01	0.4584
29	*Phragmites australis* (Cav.)Trin.ex.	−0.1151	−0.0765	1.19	1.07	0.3628
30	*Cynanchum acutum* L.	0.0990	0.0577	1.61	1.71	0.3310
31	*Lycium schweinfurthii* Dammor.	0.0307	0.0152	1.46	1.49	0.3291
32	*Cynodon dactylon (L.) Pers.*	0.1726	0.0794	1.82	1.98	0.3105
33	*Pancratium maritimum* L.	−0.3472	−0.2143	1.53	1.18	0.3327
34	*Heliotropium curassavicum* L.	−0.2229	−0.1074	1.73	1.50	0.3379
35	*Cyperus alopecuroides* Rottb.	−0.3966	−0.2171	1.66	1.27	0.4754
36	*Mesembryanthemum crystallinum* L.	0.3643	0.1278	3.43	3.79	0.2887
37	*Mesembryanthemum nodiflorum* L.	1.3183	0.4646	3.18	4.51	0.2017
38	*Solanum nigrum* L.	−0.2971	−0.1683	3.68	3.38	0.4435
39	*Saccharum spontaneum* L. Mant. Alt.	−1.1673	−0.6824	3.55	2.38	0.4483
40	*Cakile maritima* Scop.	0.4165	0.2357	3.46	3.88	0.3030
41	*Zygophyllum album L.*	0.4847	0.1834	1.67	2.16	0.1822

The Carotenoid Reflectance Indices (CRI1 and CRI2) highlighted interspecific variability in carotenoid–chlorophyll balance. High CRI values were found in *Eichhornia crassipes* (CRI1 = 9.07, CRI2 = 8.82) and *Limbarda crithmoides* (5.87), suggesting robust carotenoid activity, linked to photoprotection under high light stress [78,79]. Conversely, *Atriplex halimus* (CRI1 = 0.36, CRI2 = 0.68) displayed minimal carotenoid accumulation, consistent with its concurrently low NDVI and high MSI values, indicating vulnerability to stress.

Chlorophyll a & b indices further supported these findings. Species such as *Juncus acutus* (0.49) and *Chenopodium murale* (0.55) showed relatively high chlorophyll content, consistent with vigorous growth, whereas *Suaeda pruinosa* (0.15) and *Zygophyllum album* (0.18) had very low values, suggesting reduced photosynthetic capacity. Such differences align with known ecological strategies of halophytes, where pigment modulation contributes to salt and drought tolerance [65,82].

Overall, the integration of ARI, CRI, and chlorophyll indices indicates that while several species maintain balanced pigment profiles under Burullus Lagoon conditions, others exhibit pigment-level stress signatures. These biochemical indicators provide a complementary layer of interpretation to structural indices (e.g., NDVI, MSI), highlighting species-specific physiological strategies for stress resilience.

Pigment-related indices also revealed distinct physiological strategies among the surveyed species. High CRI values in *Eichhornia crassipes* reflected its efficient photoprotective capacity and rapid biomass accumulation typical of floating hydrophytes. In contrast, the markedly elevated ARI values observed in *Spergularia marina* and *Mesembryanthemum nodiflorum* indicated enhanced anthocyanin production, a known mechanism supporting tolerance to salinity and intense radiation in coastal and saltmarsh habitats. Species such as *Suaeda pruinosa* and *Zygophyllum album* exhibited notably low chlorophyll concentrations, suggesting limited photosynthetic capacity and resource-conservative strategies characteristic of stress-tolerant halophytes. Together, these pigment-based responses provide a coherent ecological interpretation of species performance across the Burullus Lagoon environment.

Table 9 compiles red-edge indices, which provide sensitive indicators of chlorophyll concentration and pigment dynamics across species. The Red Edge Normalized Difference Vegetation Index (RENDVI) was effective in capturing chlorophyll content variation across species (Table 9). Based on Gitelson et al. (2001), healthy vegetation is typically associated with RENDVI values between 0.2–0.9. In our dataset, most species (e.g., *Chenopodium murale*, 0.55; *Echinochloa stagnina*, 0.50) fell within this range, indicating adequate chlorophyll and canopy structure. However, species such as *Suaeda pruinosa* (0.15), *Tamarix nilotica* (0.18), and *Spergularia marina* (0.14) showed values below 0.2, reflecting reduced photosynthetic activity and pigment degradation [79,83].

**Table 9:** Red-edge vegetation indices (RENDVI, MRENDVI, MRESR, and REPI).

No.	Plant Species	RENDVI	MRENDVI	MRESR	REPI1	REPI2
1	*Halocnemum strobilceum* (Pallas) M. Bieb.	0.3544	0.1300	3.35	0.2827	689.44
2	*Suaeda pruinosa* Lange.	0.1516	−0.1119	1.57	0.3532	671.61
3	*Lactuca serriola* L.	0.4288	0.2030	3.96	0.3502	690.95
4	*Juncus acutus* L.	0.4852	0.3697	4.01	0.2661	692.97
5	*Atriplex portulacoides* L.	0.2819	−0.0698	2.66	0.4079	687.16
6	*Atriplex halimus* L.	0.1367	−0.4139	1.74	0.5344	687.15
7	*Chenopodium murale* L.	0.5464	0.3779	6.13	0.3134	694.38
8	*Rumex dentatus* L.	0.4679	0.3199	4.04	0.2849	692.68
9	*Limbarda crithmoides* (L.) Dumort.	0.2871	0.1114	2.12	0.3550	679.77
10	*Arthrocnemum macrostachyum* (Moric.) K. Koch	0.3316	0.1642	2.99	0.2209	690.05
11	*Atriplex semibaccata* R.Br.	0.2217	−0.0712	2.07	0.3529	683.24
12	*Tamarix nilotica* (Ehrenb.) Bunge.	0.1854	−0.0444	2.06	0.2218	683.27
13	*Spergularia marina* (L.) Griseb.	0.1428	0.0033	1.45	0.2548	664.19
14	*Echinochloa stagnina* (Retz.) P. Beauv.	0.5030	0.3153	6.97	0.2762	694.89
15	*Sonchus oleraceus* L.	0.3618	0.1937	3.12	0.2523	689.71
16	*Eichhornia crassipes* (C. Mart.) Solms.	0.4754	0.3857	3.33	0.3108	690.58
17	*Alhagi graecorum* Boiss.	0.3343	0.1351	2.97	0.2683	687.81
18	*Symphyotrichum squamatum* (Spren.) Nesom.	0.4871	0.3363	5.21	0.2451	693.80
19	*Polygonium equistiform* Sm.	0.4246	0.1763	3.91	0.3917	691.10
20	*Suaeda vera* Forssk. ex J.F. Gmel.	0.4540	0.2275	4.52	0.3417	691.66
21	*Persicaria salicifolia* (Willd) Assenov.	0.4073	0.2109	3.29	0.3471	690.12
22	*Typha domingensis* (Pers.) Poir.ex Steud.	0.3853	0.1360	3.11	0.4147	688.52
23	*Juncus rigidus* Desf.	0.2863	0.1390	2.63	0.1792	686.08
24	*Bassia indica* (Wight) A.J.Scott.	0.3588	0.1128	4.14	0.2696	691.87
25	*Pluchea dioscoridis* (L.) DC.	0.3400	0.1332	2.65	0.3368	685.39
26	*Zygophyllum aegyptium* Hosny.	0.3150	0.1336	2.49	0.2803	684.12
27	*Malva parviflora* L.	0.2082	0.0136	1.91	0.2601	681.16
28	*Suaeda maritima* (L.) Dumort.	0.4584	0.2452	4.88	0.3134	692.72
29	*Phragmites australis* (Cav.)Trin.ex.	0.3628	0.0486	3.52	0.4042	688.85
30	*Cynanchum acutum* L.	0.3310	0.0394	3.03	0.3653	688.25
31	*Lycium schweinfurthii* Dammor.	0.3291	0.0704	3.12	0.3184	689.78
32	*Cynodon dactylon (L.) Pers.*	0.3105	0.0809	2.78	0.2958	687.69
33	*Pancratium maritimum* L.	0.3327	−0.0201	3.38	0.3825	687.92
34	*Heliotropium curassavicum* L.	0.3379	0.0486	3.76	0.2980	688.09
35	*Cyperus alopecuroides* Rottb.	0.4754	0.2331	6.36	0.3292	695.06
36	*Mesembryanthemum crystallinum* L.	0.2887	0.1450	2.32	0.2160	682.93
37	*Mesembryanthemum nodiflorum* L.	0.2017	0.0406	1.78	0.2225	672.25
38	*Solanum nigrum* L.	0.4435	0.2578	3.84	0.3285	692.38
39	*Saccharum spontaneum* L. Mant. Alt.	0.4483	0.2791	3.67	0.3334	690.57
40	*Cakile maritima* Scop.	0.3030	0.1370	2.21	0.3252	680.22
41	*Zygophyllum album* L.	0.1822	−0.0639	1.86	0.2624	676.47

The Modified Red Edge NDVI (MRENDVI) was designed to enhance sensitivity to subtle chlorophyll differences [48]. Thirteen species (e.g., *Juncus acutus*, 0.37; *Chenopodium murale*, 0.38; *Symphyotrichum squamatum*, 0.34) displayed positive MRENDVI values within the 0.2–0.7 healthy range, confirming good canopy condition. Negative values in *Atriplex halimus* (–0.41) and *Atriplex portulacoides* (–0.07) suggest reduced pigment absorption, consistent with stress-related physiological adjustments under salinity. These patterns are consistent with the findings of [82],who highlighted the sensitivity of MRENDVI to reduced chlorophyll under stress conditions.

The Modified Red Edge Simple Ratio (MRESR or mSR705) further differentiated taxa. According to Gitelson & Merzlyak [65], values between 2–8 are indicative of healthy photosynthetic function. Higher values in *Echinochloa stagnina* (6.97) and *Cyperus alopecuroides* (6.36) indicate strong photosynthetic capacity, whereas species such as, *Spergularia marina* (1.45), *Malva parviflora* (1.91), and *Mesembryanthemum nodiflorum* (1.78) fell below 2, highlighting reduced photosynthetic efficiency.

The Red Edge Position Index (REPI) quantifies shifts in the red edge inflection point, directly linked to leaf chlorophyll content [83]. In this study, REPI values ranged between 664–695 nm, with *Spergularia marina* showing the lowest value (664 nm) and *Cyperus alopecuroides* the highest (695 nm). Lower REPI values (e.g., *Spergularia marina*, 664 nm) indicate reduced chlorophyll content, whereas higher REPI values (e.g., *Cyperus alopecuroides*, 695 nm) correspond to greater chlorophyll accumulation and photosynthetic potential.

Taken together, the red-edge indices provide ecologically meaningful differentiation among species. Chlorophyll-rich species such as *Chenopodium murale* and *Echinochloa stagnina* demonstrated consistently high red-edge values, reflecting vigorous growth and efficient pigment functioning. In contrast, halophytic taxa such as *Suaeda pruinosa* and *Atriplex halimus* exhibited reduced red-edge responses, highlighting stress-linked pigment reduction consistent with their drought- and salinity-tolerant strategies. These species-level patterns illustrate how red-edge indices capture variations in photosynthetic efficiency, pigment regulation, and adaptive responses across the Burullus wetland [82] (Table 9).

## 4. Discussion

Moreover, the ecological patterns observed in the floristic and life-form analyses provide a broader context for interpreting the spectral behavior of the surveyed taxa. The dominance of therophytes and chamaephytes across much of the Burullus wetland reflects a community structure shaped by recurrent disturbances, seasonal water fluctuations, and spatially heterogeneous salinity gradients [69,72,73]. These functional groups typically exhibit rapid life cycles, shallow rooting systems and opportunistic resource use, traits that are consistent with their moderate chlorophyll signals and relatively variable NDVI values. In contrast, the presence of perennial halophytic shrubs within the central and eastern basins—such as *Atriplex*, *Tamarix* and *Suaeda*—indicates long-term adaptation to stable yet stressful saline regimes, which aligns with the lower photosynthetic activity and higher stress-related spectral indices reported in this study. Chorological patterns further support this ecological interpretation: the predominance of Saharo-Arabian and Mediterranean–Irano-Turanian elements suggests that many of the dominant taxa are pre-adapted to arid, saline and nutrient-poor conditions, explaining their conservative water-use traits and distinctive red-edge signatures. Spatial differences between northern, southern and islet habitats also reinforce the role of environmental heterogeneity in driving species assemblages, with hydrophytes occupying deeper, nutrient-rich waters and generating high-chlorophyll spectral profiles, while xerohalophytes colonize elevated saline margins where spectral stress indicators increase noticeably [74].

Spectral indices extend beyond physiological assessment to provide clear ecological meaning for species strategies and Wetland dynamics. For example, species with low NDVI and high MSI values such as *Atriplex halimus* and *Tamarix nilotica* reflect stress-tolerant halophytic strategies that persist under salinity and water deficit, contributing to patchy vegetation cover and reduced net primary productivity [71]. Conversely, the very high NDVI recorded for *Eichhornia crassipes* illustrates vigorous growth, which ecologically translates to invasive dominance that suppresses native biodiversity and alters ecosystem functioning. Pigment indices such as ARI and CRI further highlight adaptive mechanisms, where anthocyanin accumulation (*Suaeda monoica*) or carotenoid investment (*Mesembryanthemum nodiflorum*) indicate photoprotective and antioxidant roles under extreme radiation and salinity [84]. Similarly, red-edge indices (RENDVI, REPI) capture subtle variation in chlorophyll content and canopy structure, which ecologically corresponds to differences in photosynthetic efficiency, competitive ability, and community assembly. Collectively, these results emphasize that spectral indices not only quantify plant condition but also serve as proxies for ecological strategies, stress tolerance, and species interactions within the Burullus Lagoon ecosystem. Furthermore, linking these spectral patterns with species-level ecological traits clarifies how dominant taxa maintain physiological performance under contrasting habitat conditions—for instance, hydrophytes with high chlorophyll and carotenoid signals (e.g., *Eichhornia crassipes*) exhibit competitive dominance, while halophytes with reduced NDVI and elevated stress indices (e.g., *Atriplex halimus*, *Tamarix nilotica*) reflect resource-conservative strategies adapted to salinity and water limitation. Recent hyperspectral studies also confirm that integrating red-edge and pigment indices improves the detection of subtle physiological stress under salinity and drought, supporting species-level discrimination in wetland ecosystems [79,82].

## 5. Conclusion

This study developed a spectral library for Burullus Lagoon by integrating floristic surveys with hyperspectral measurements, covering 41 species across key habitats. The results demonstrate the utility of hyperspectral indices for assessing plant health, stress responses, and ecological dynamics in wetland ecosystems. The spectral library provides a valuable baseline for monitoring vegetation changes and supports conservation and sustainable management of this Ramsar-listed wetland. Future applications can build on this dataset to enhance wetland monitoring under increasing anthropogenic and climate pressures.

## Supporting information

S1 File Supplementary data. (DOCX)

S2 File Supplementary data. (XLSX)
