# Supplementary material for: Field-based hyperspectral characterization of wetland plant diversity and vitality in Burullus Lagoon (Nile Delta, Egypt)
Source: PLoS One. 2026 Mar 4;21(3):e0341891. doi: 10.1371/journal.pone.0341891 (PMC12959720; doi:10.1371/journal.pone.0341891)

**Table S1.** Coordinates of sampling sites within the Burullus Lake

| **Site No.** | **Habitat** | **Coordinates** | | | | | |
| --- | --- | --- | --- | --- | --- | --- | --- |
|  |  | **Latitude (N)** | | | **Longitude (E)** | | |
| 1 | **Lake Shore** | 31 | 23 | 6.19 | 30 | 36 | 17.14 |
| 2 |  | 31 | 23 | 41.74 | 30 | 35 | 34.59 |
| 3 |  | 31 | 24 | 27.00 | 30 | 34 | 48.00 |
| 4 |  | 31 | 25 | 21.60 | 30 | 34 | 7.81 |
| 5 |  | 31 | 25 | 27.31 | 30 | 33 | 23.24 |
| 6 |  | 31 | 28 | 44.28 | 30 | 32.3 | 21.78 |
| 7 |  | 31 | 30 | 24.86 | 30 | 31.6 | 20.32 |
| 8 |  | 31 | 32 | 19.88 | 30 | 30.9 | 18.87 |
| 9 |  | 31 | 33 | 6.09 | 30 | 30.2 | 17.41 |
| 10 |  | 31 | 34 | 13.27 | 30 | 29.5 | 15.95 |
| 11 |  | 31 | 34 | 46.12 | 30 | 28.8 | 14.49 |
| 12 |  | 31 | 34 | 24.08 | 31 | 1 | 26.28 |
| 13 |  | 31 | 33 | 26.62 | 31 | 4 | 30.56 |
| 14 |  | 31 | 31 | 34.66 | 31 | 4 | 49.21 |
| 15 |  | 31 | 30 | 59.58 | 31 | 2 | 0.94 |
| 16 |  | 31 | 26 | 58.33 | 30 | 59 | 1.07 |
| 17 |  | 31 | 24 | 58.12 | 30 | 53 | 43.78 |
| 18 |  | 31 | 25 | 22.92 | 30 | 51 | 15.94 |
| 19 |  | 31 | 24 | 29.65 | 30 | 47 | 27.38 |
| 20 |  | 31 | 24 | 18.50 | 30 | 45 | 14.34 |
| 21 |  | 31 | 23 | 53.17 | 30 | 40 | 53.71 |
| 22 |  | 31 | 22 | 53.80 | 30 | 37 | 6.68 |
| 23 | **Islets** | 31 | 32 | 2.24 | 30 | 58 | 15.16 |
| 24 |  | 31 | 32 | 4.06 | 30 | 58 | 53.70 |
| 25 |  | 31 | 26 | 48.25 | 30 | 49 | 44.55 |
| 26 |  | 31 | 26 | 55.74 | 30 | 50 | 35.61 |
| 27 |  | 31 | 27 | 31.77 | 30 | 51 | 36.88 |
| 28 |  | 31 | 26 | 16.74 | 30 | 47 | 53.43 |
| 29 |  | 31 | 25 | 35.21 | 30 | 48 | 2.91 |
| 30 |  | 31 | 28 | 6.53 | 30 | 48 | 4.40 |
| 31 |  | 31 | 30 | 8.13 | 30 | 51 | 30.30 |
| 32 |  | 31 | 25 | 2.10 | 30 | 40 | 16.89 |
| 33 |  | 31 | 24 | 45.05 | 30 | 40 | 58.92 |
| 34 | **Open water** | 31 | 25 | 17.64 | 30 | 38 | 29.34 |
| 35 |  | 31 | 25 | 4.77 | 30 | 39 | 0.23 |
| 36 |  | 31 | 26 | 23.02 | 30 | 42 | 30.32 |
| 37 |  | 31 | 25 | 24.51 | 30 | 43 | 41.40 |
| 38 |  | 31 | 25 | 7.07 | 30 | 45 | 32.45 |
| 39 |  | 31 | 26 | 18.43 | 30 | 50 | 50.21 |
| 40 |  | 31 | 30 | 58.29 | 30 | 51 | 19.54 |
| 41 |  | 31 | 28 | 11.68 | 30 | 50 | 20.44 |
| 42 |  | 31 | 33 | 12.75 | 30 | 57 | 31.23 |
| 43 |  | 31 | 33 | 37.56 | 31 | 0 | 0.47 |
| 44 |  | 31 | 33 | 12.39 | 31 | 1 | 53.67 |

**Figures S1.** **Spectral reflectance pattern for plant species.**


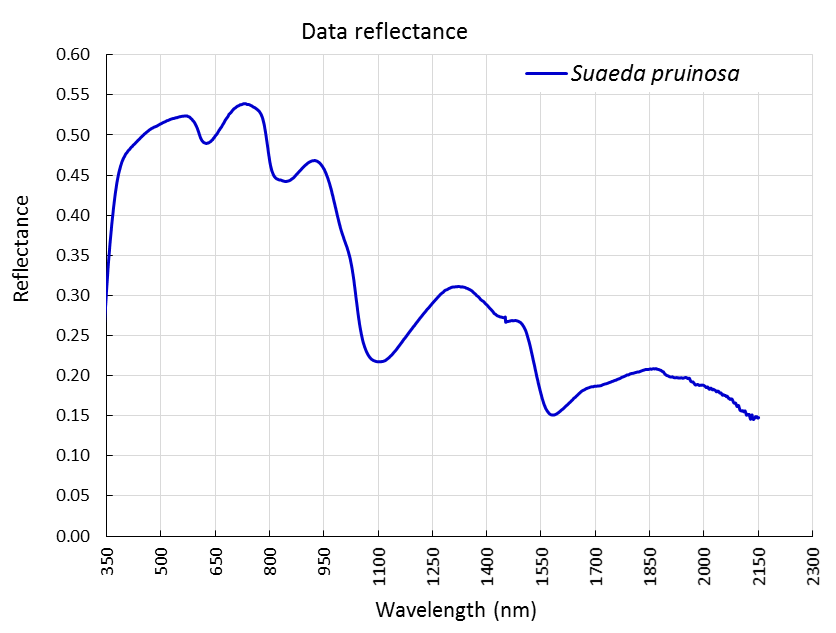

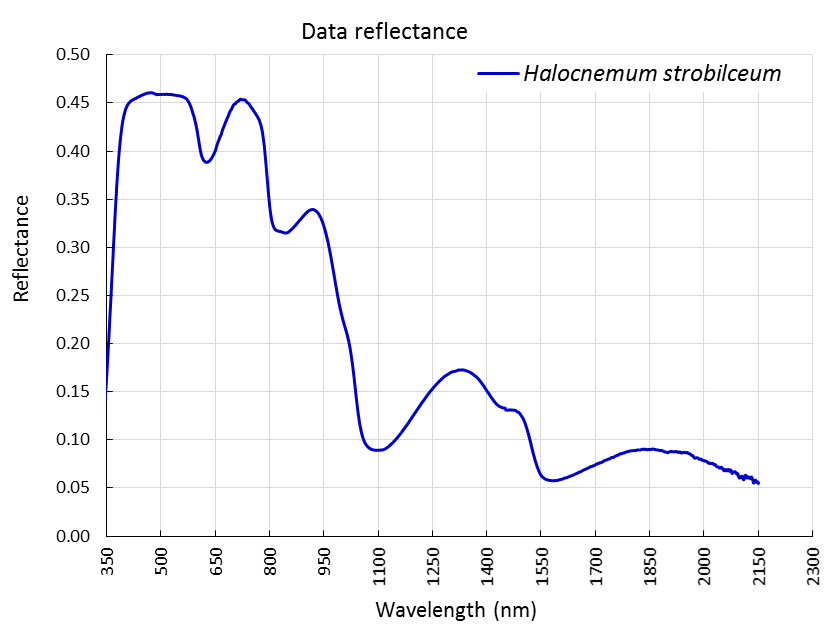

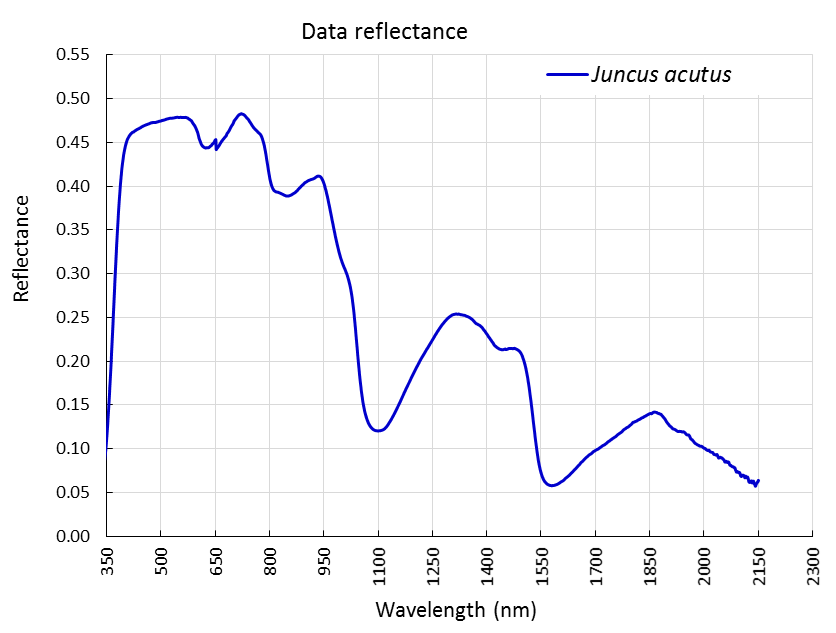

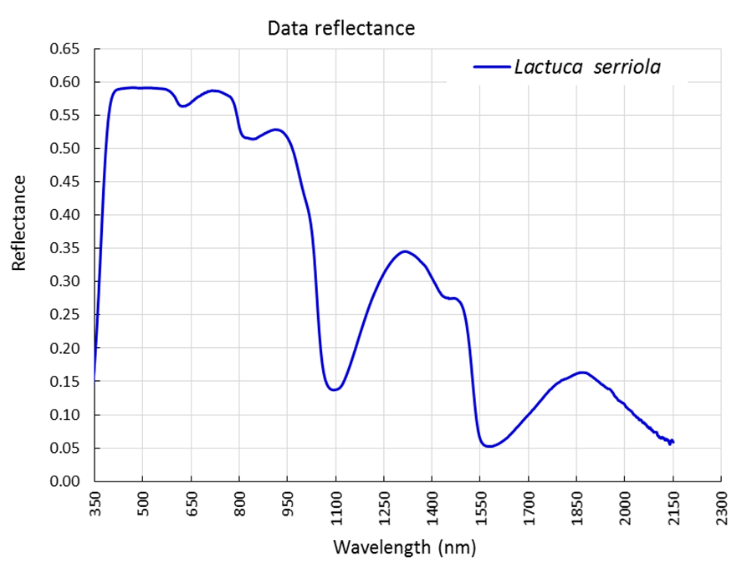


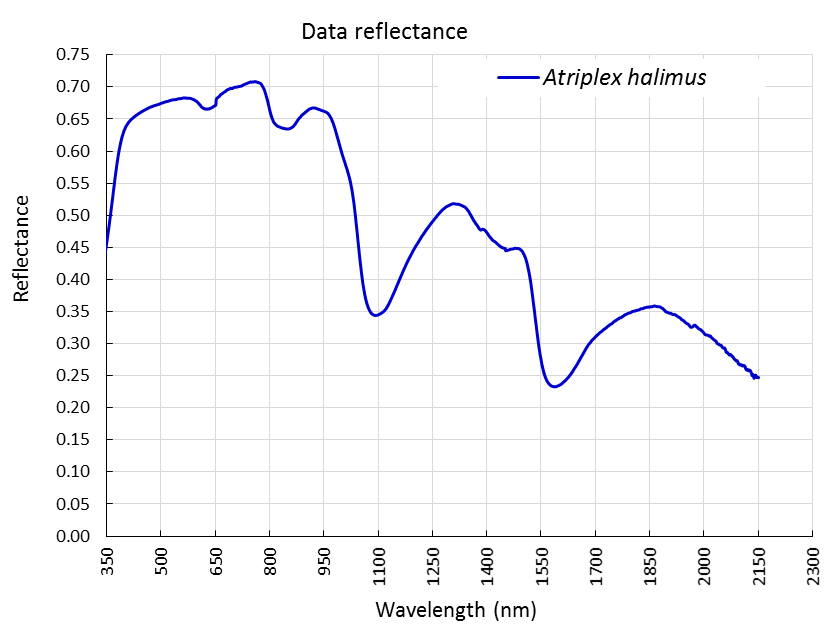

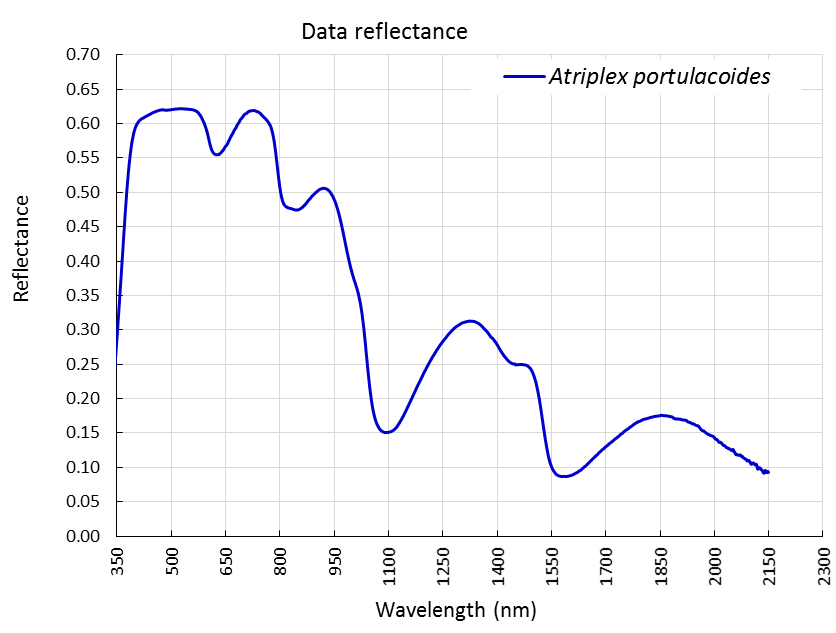


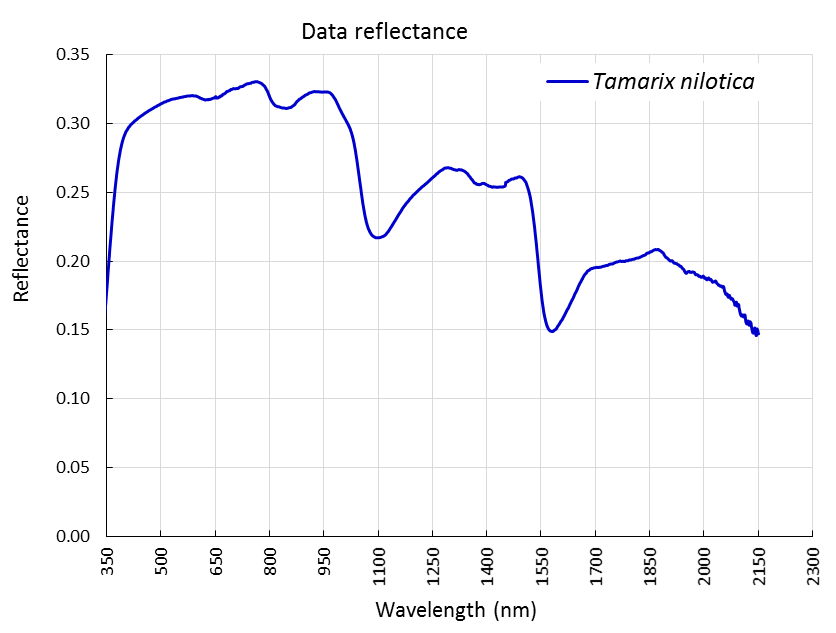

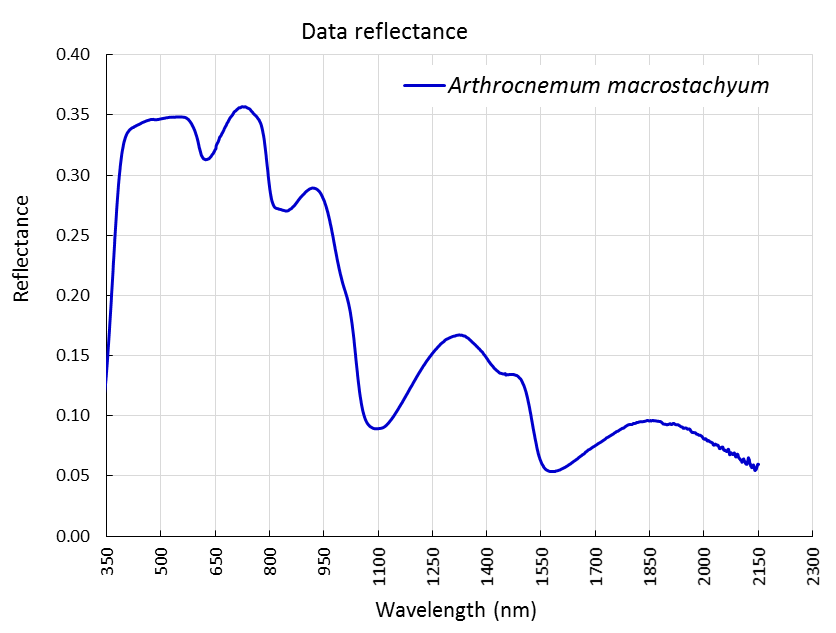

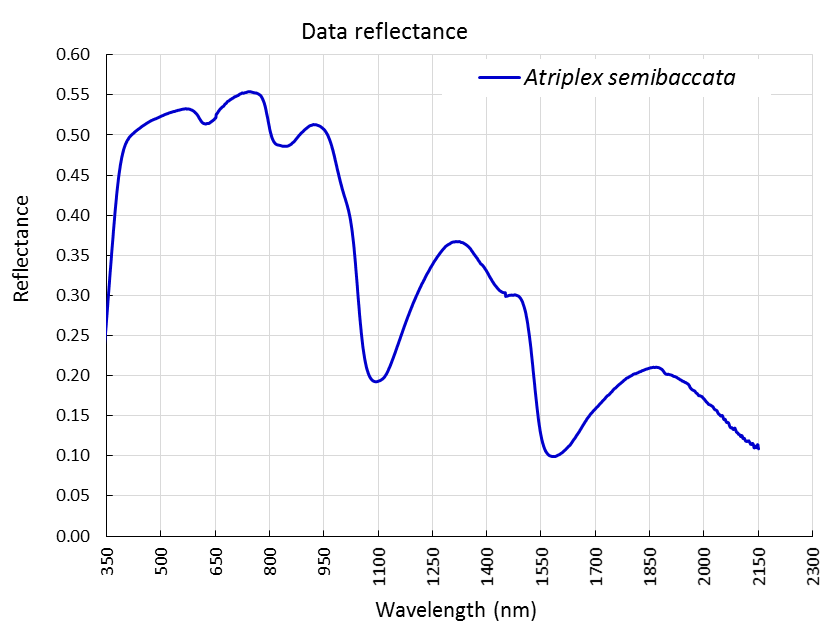

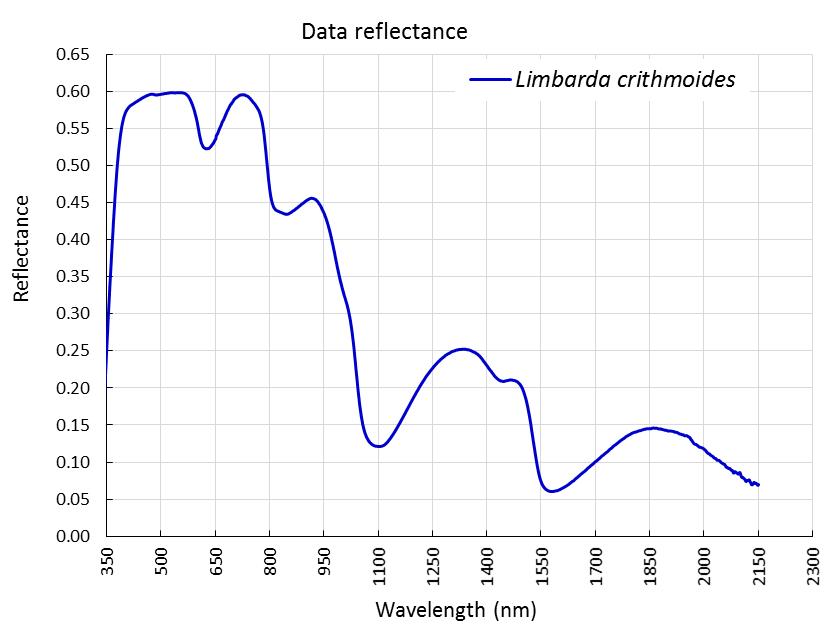

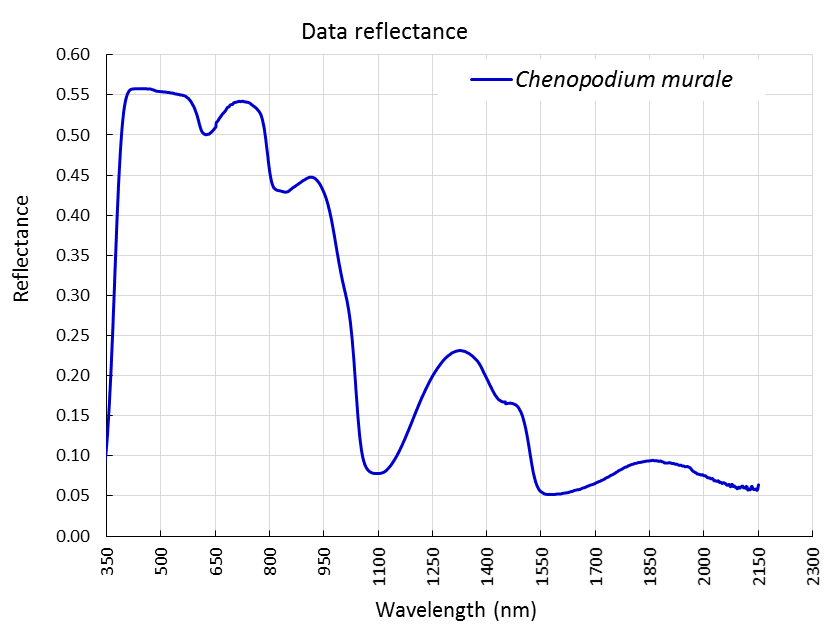

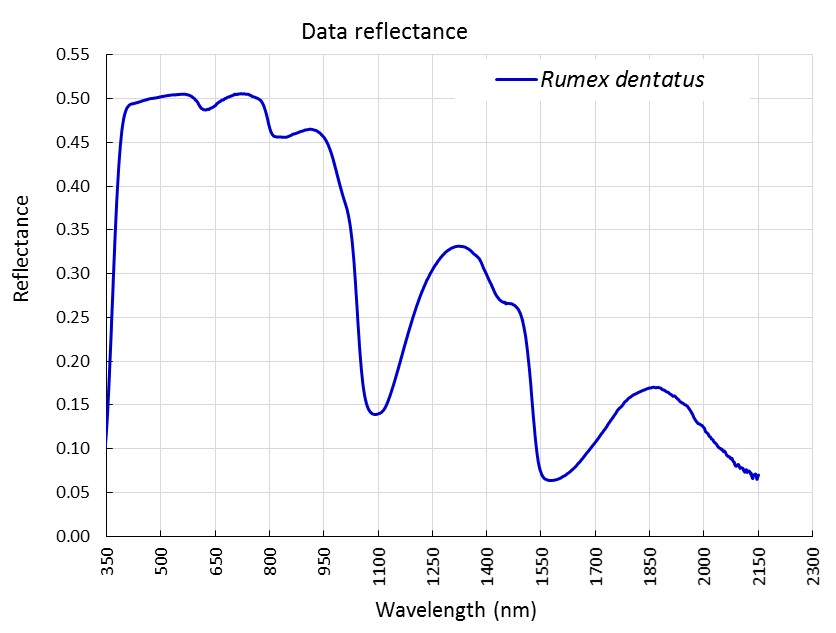


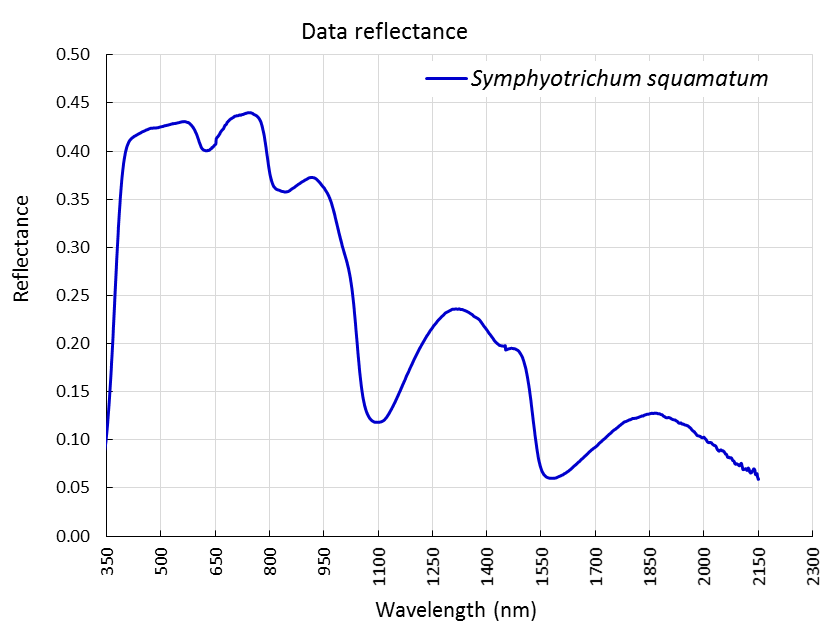

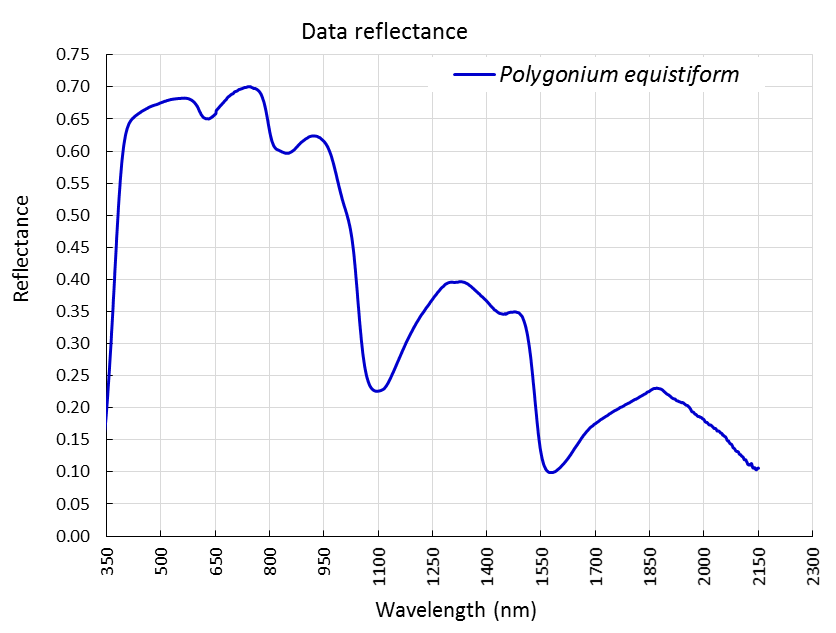

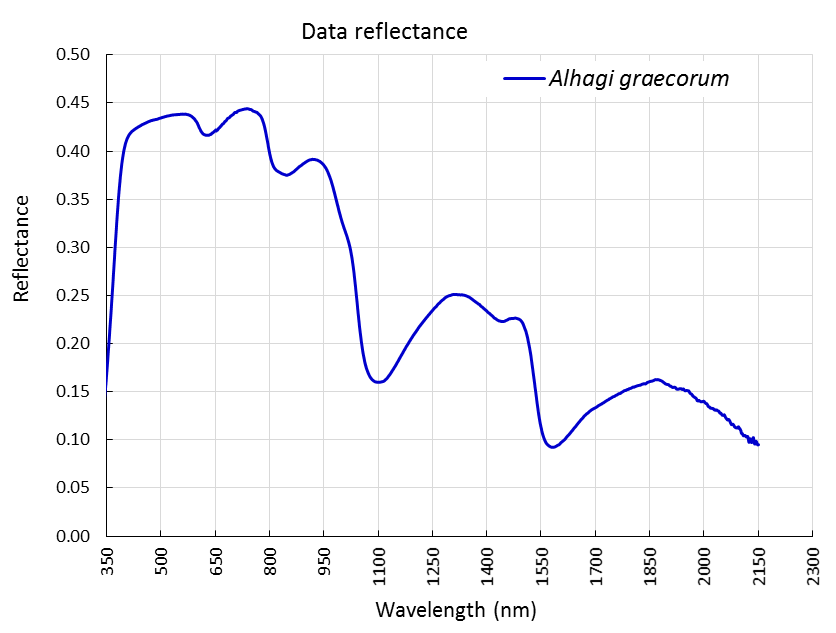

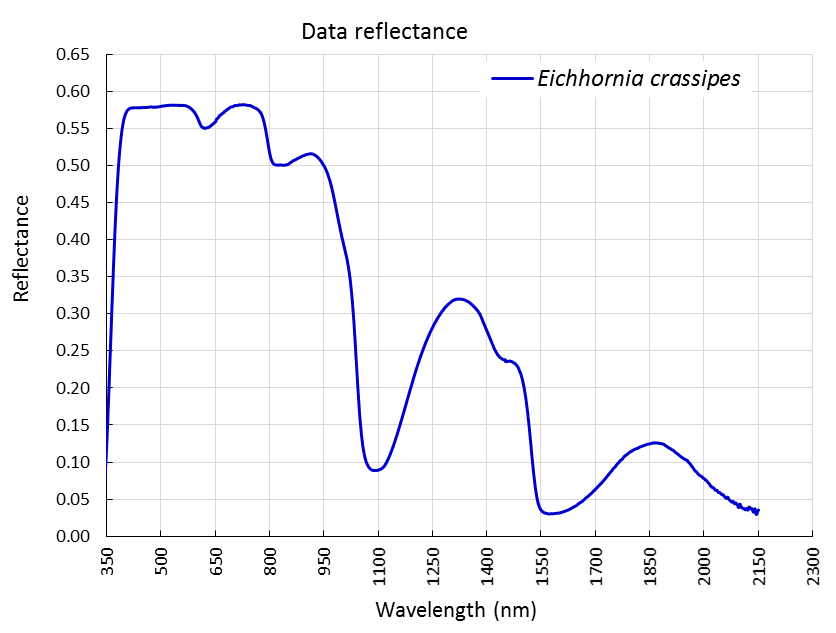

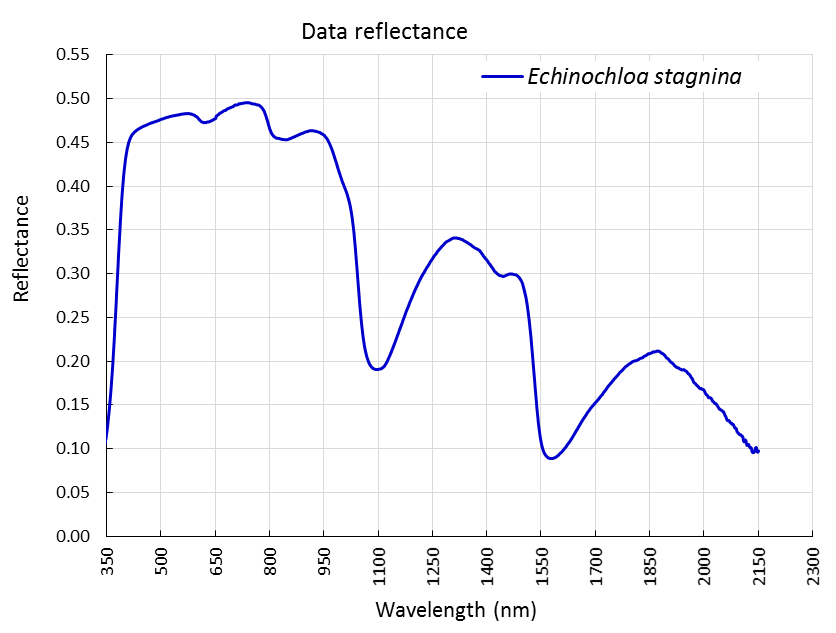

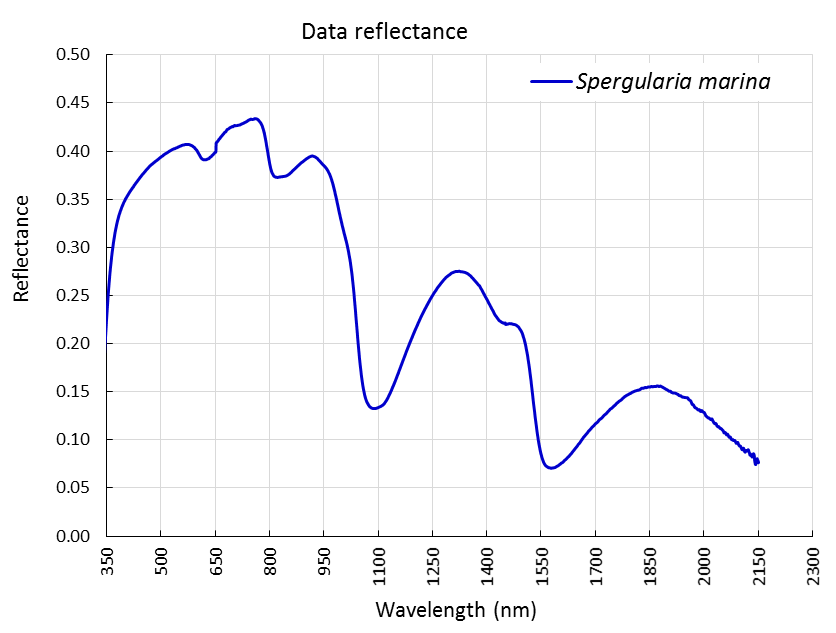


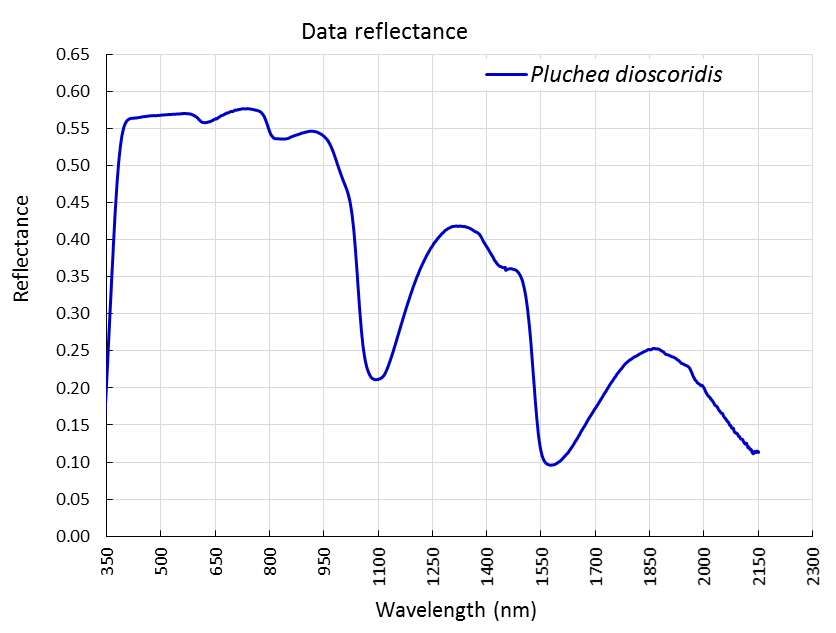

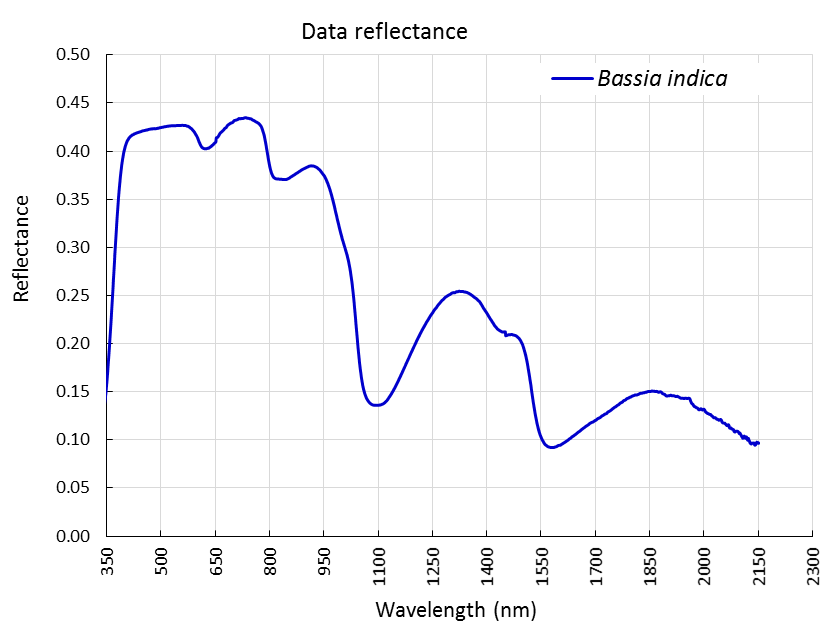

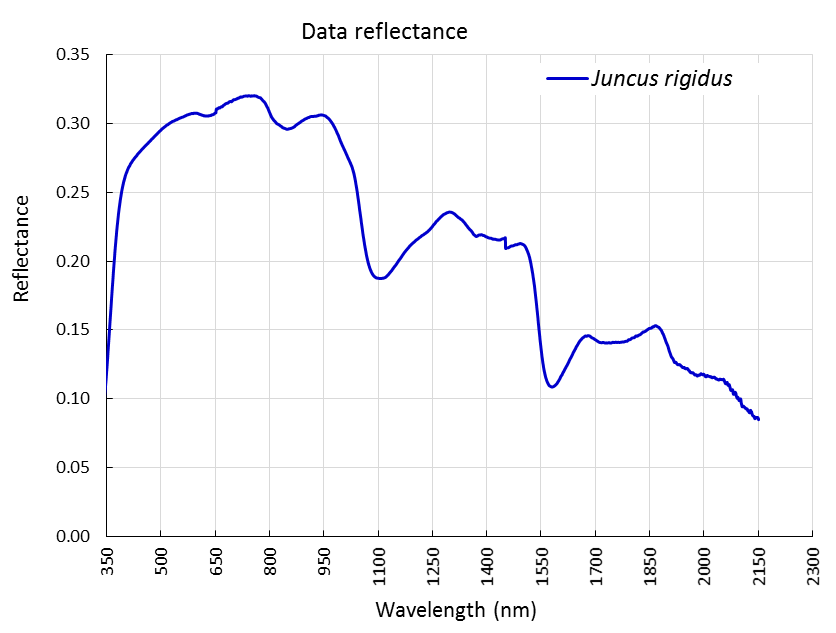

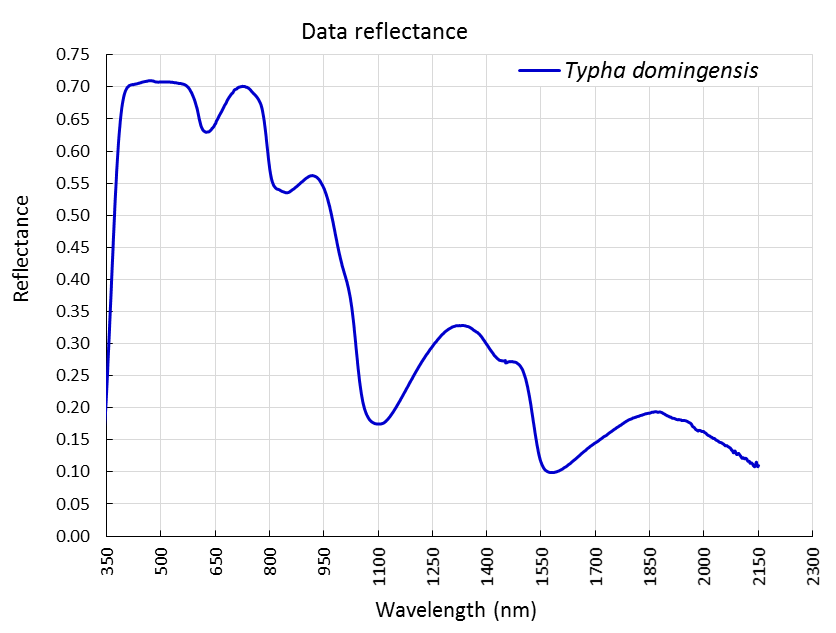

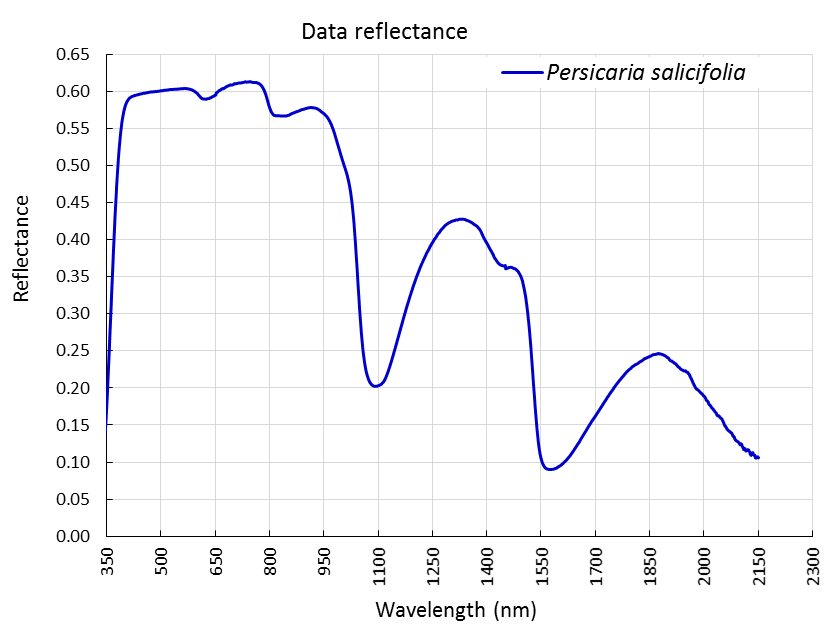

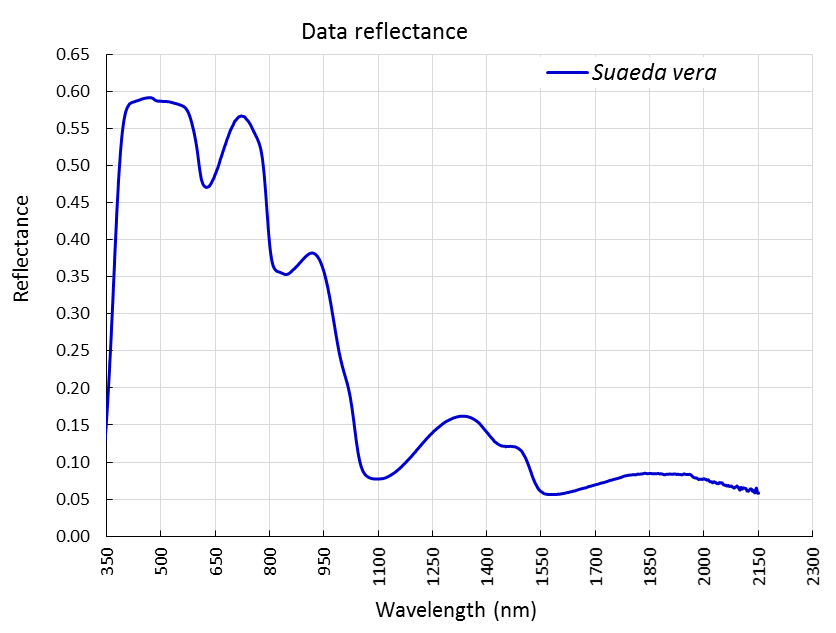


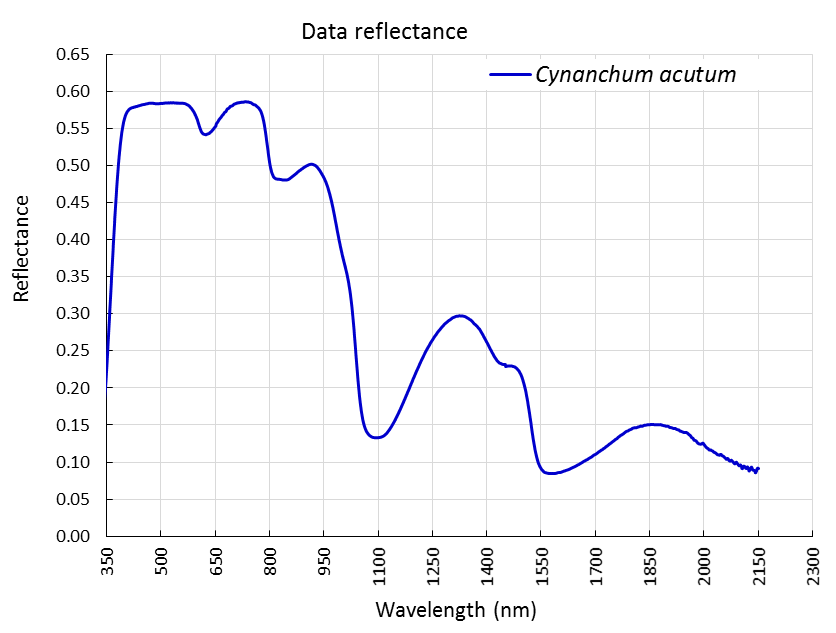

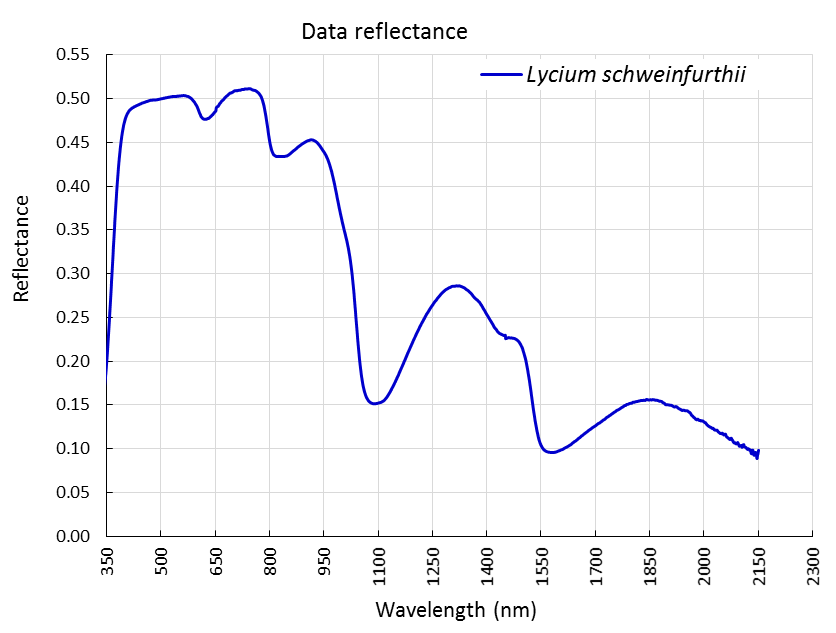

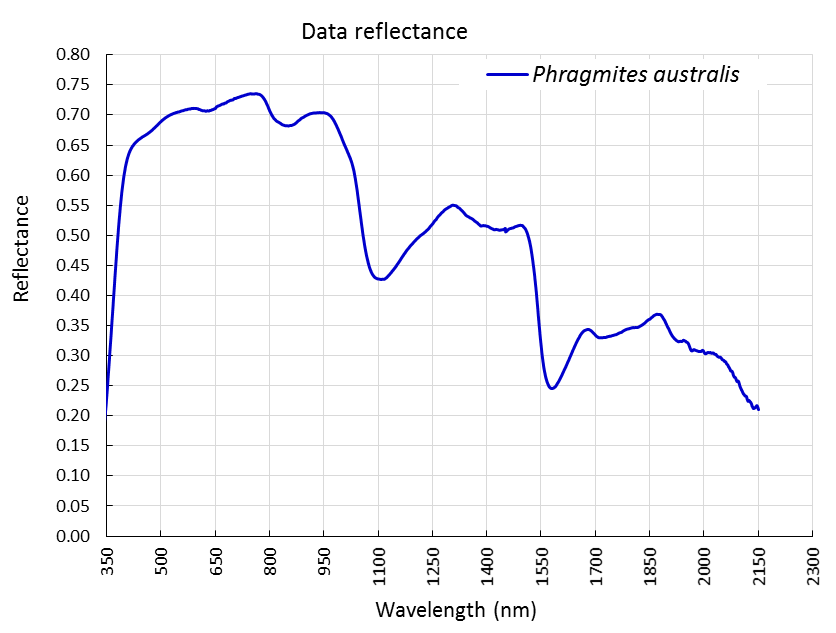

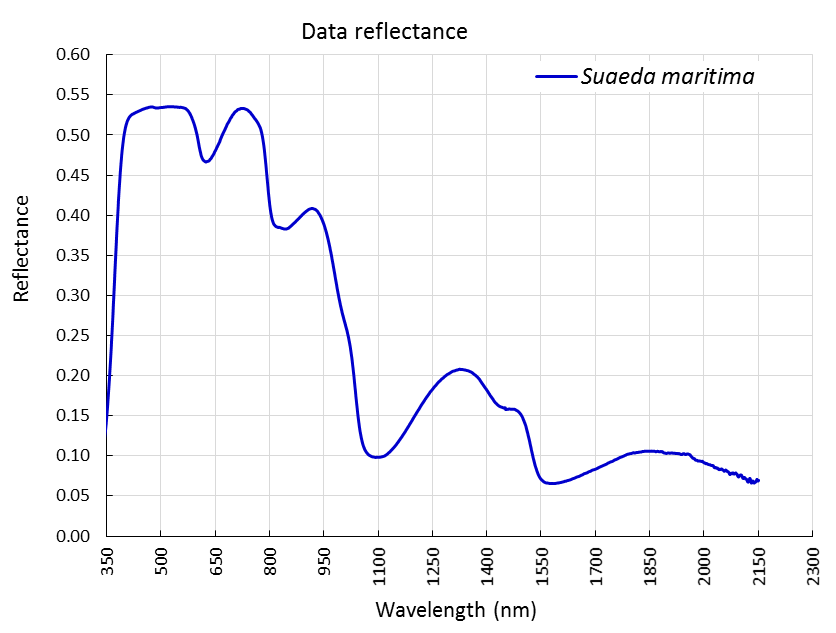

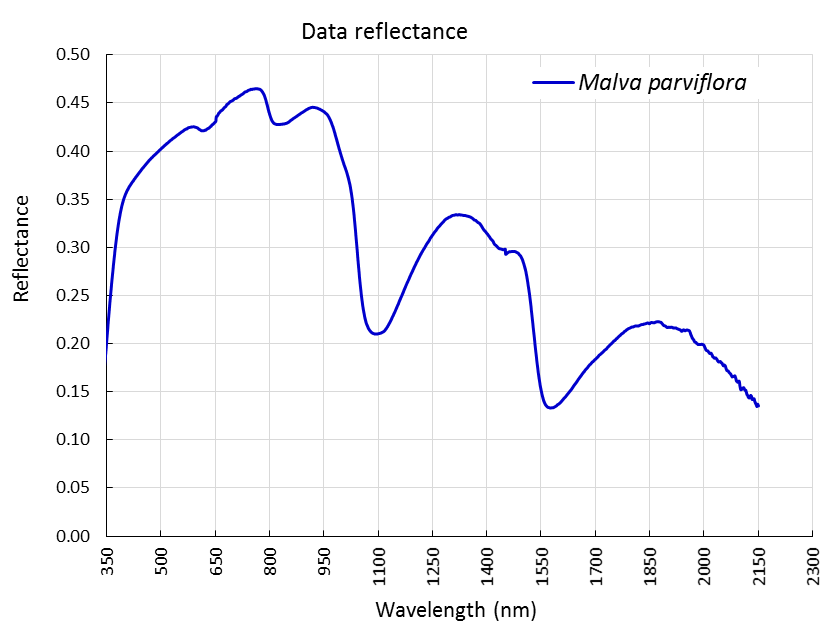

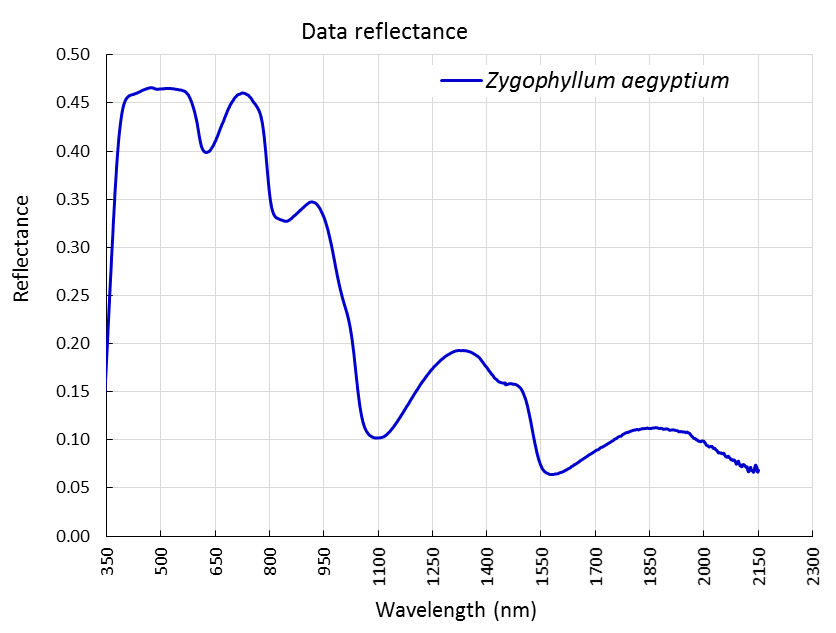


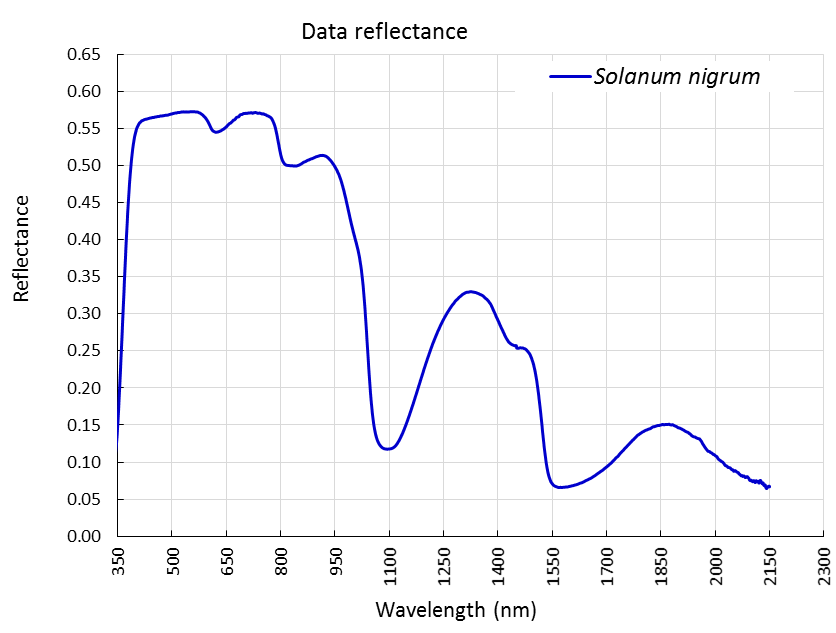

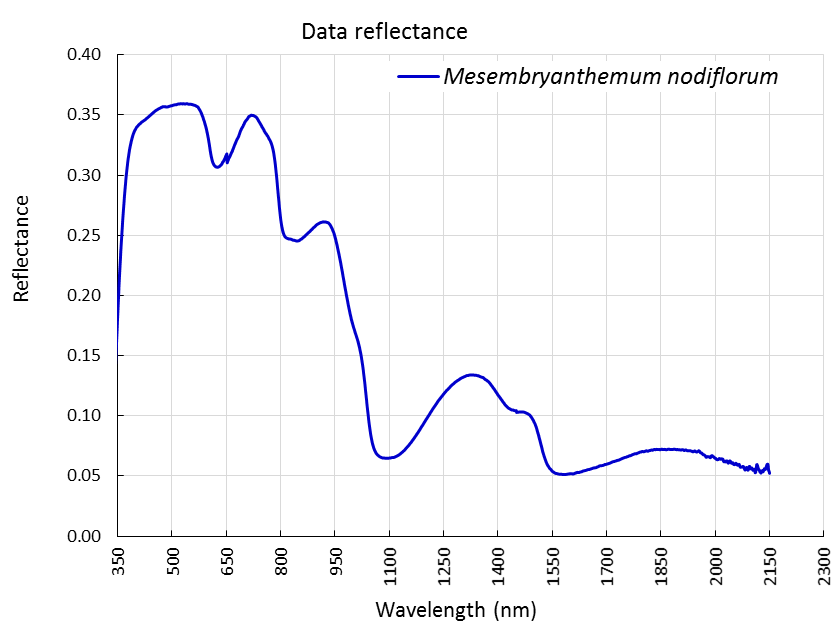

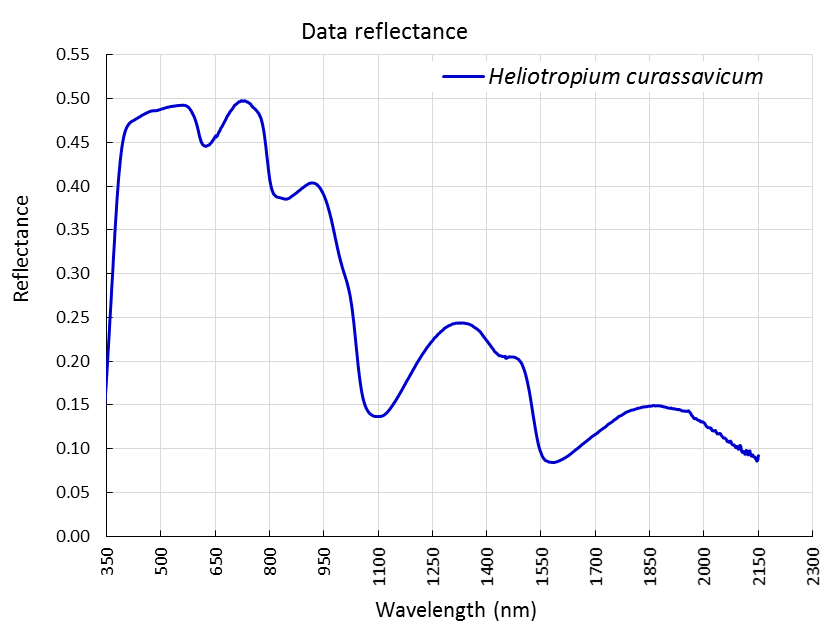

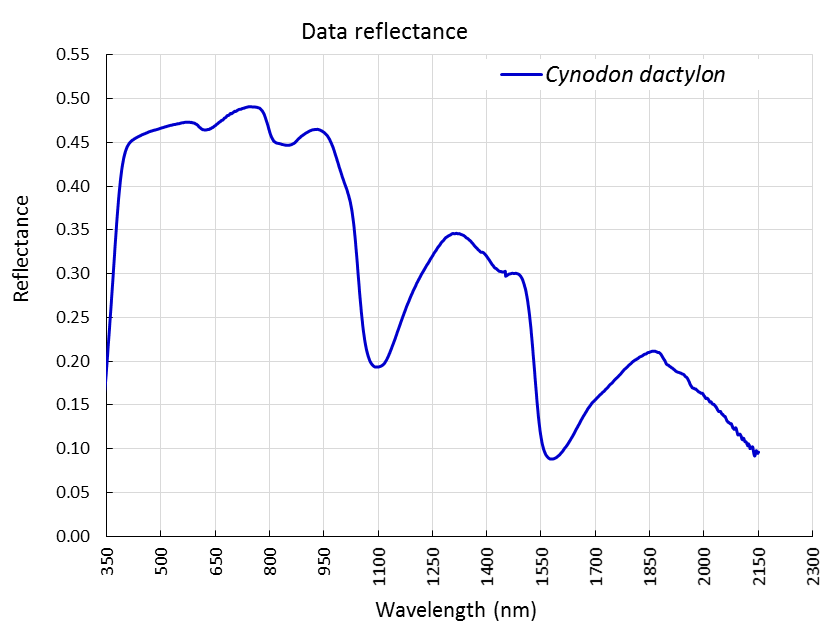

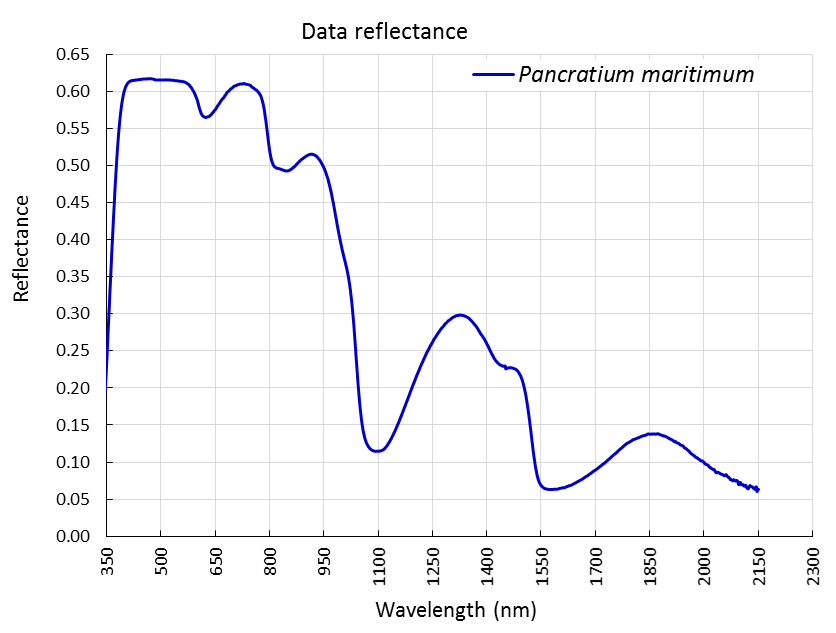

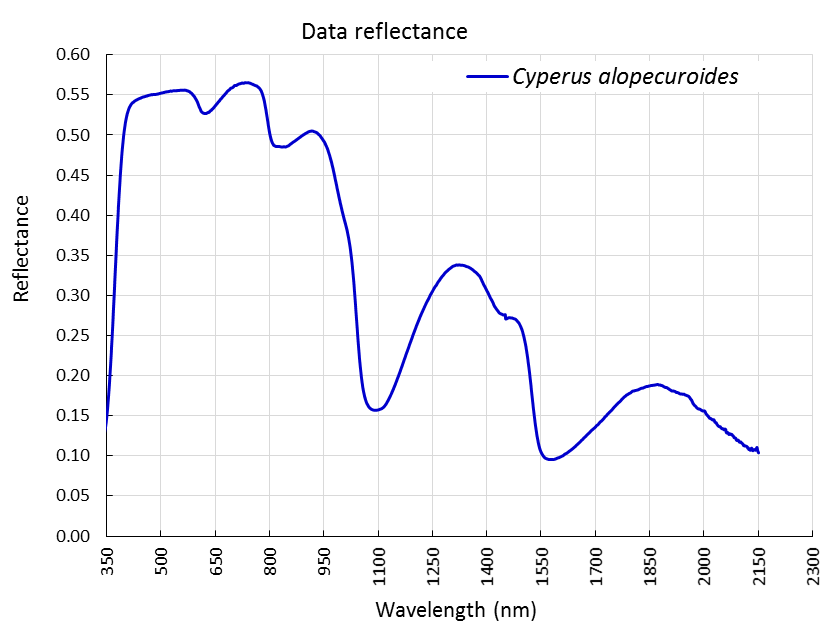


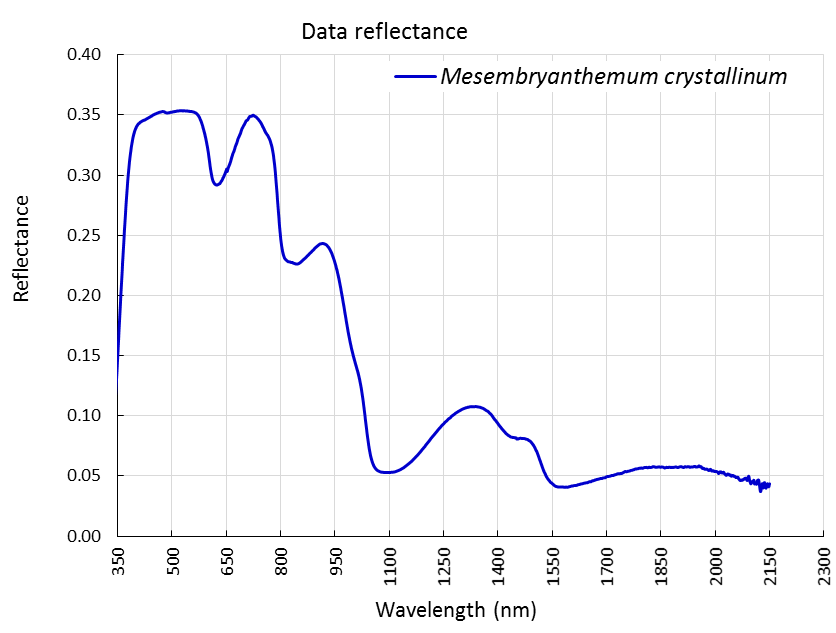

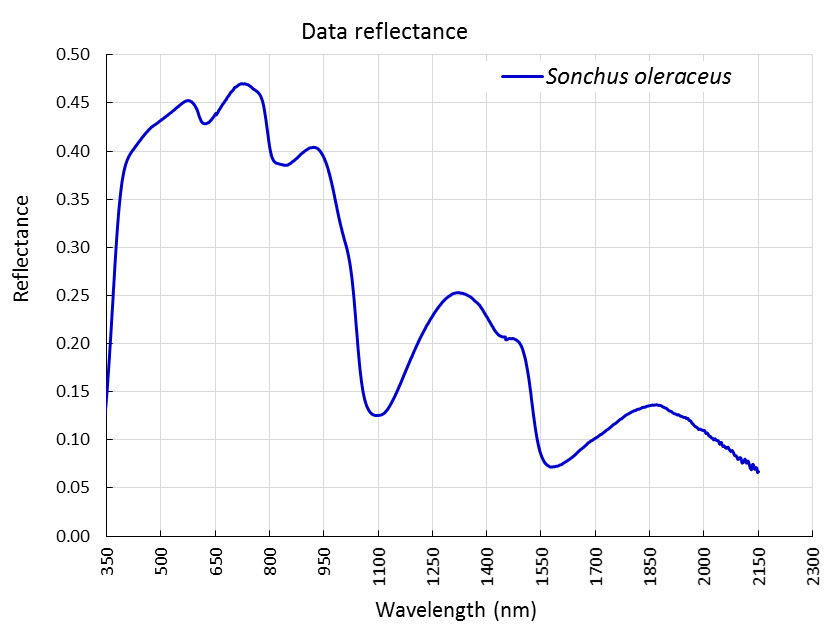

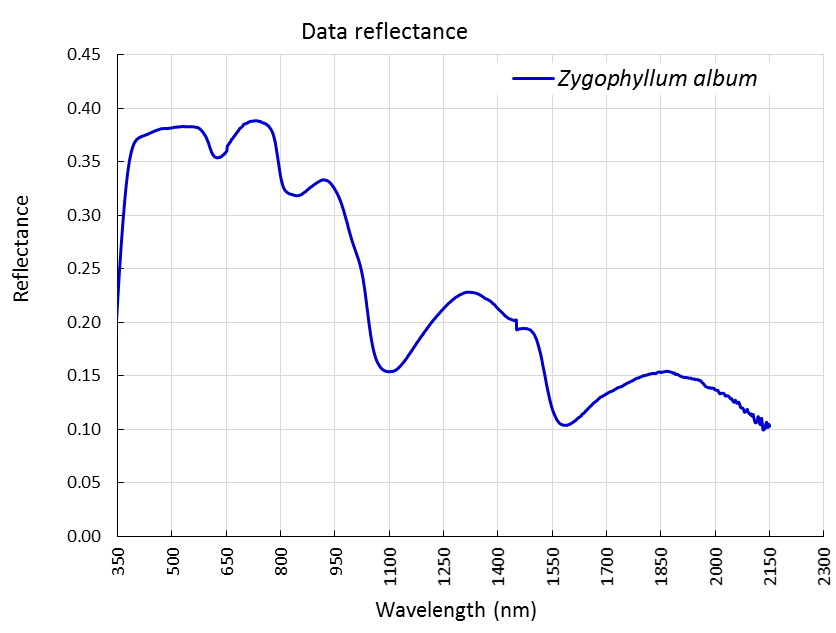

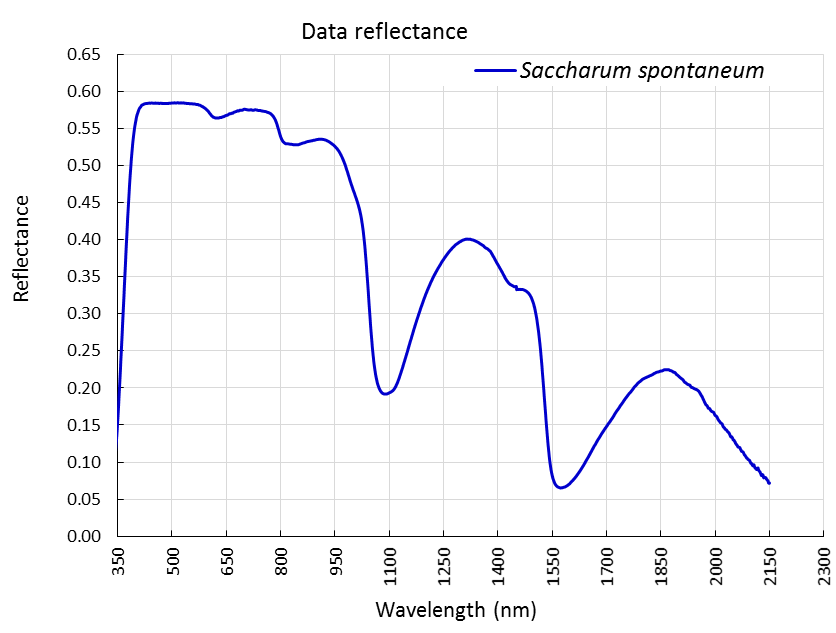

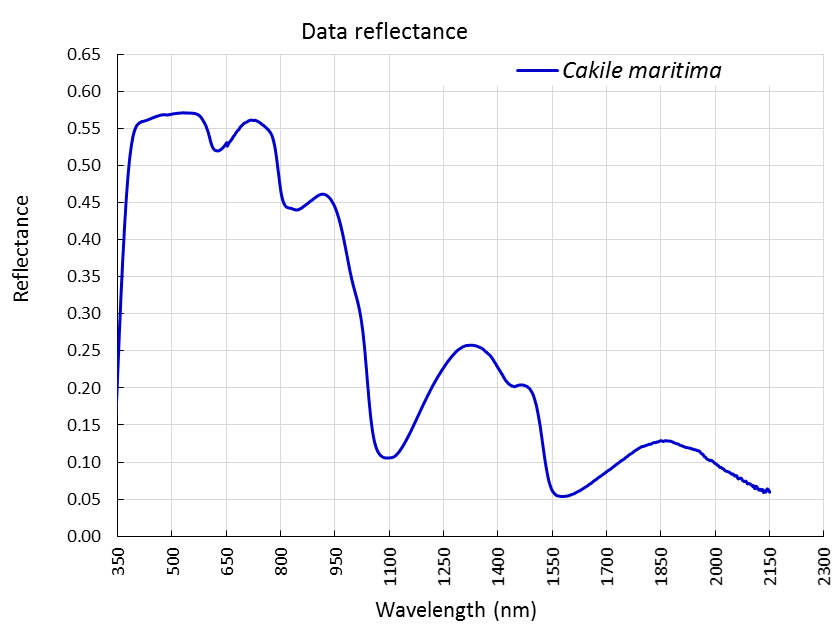

Supplement: S1 File — (DOCX) [file pone.0341891.s001.docx]
